# Protein engineering of a nanoCLAMP antibody mimetic scaffold as a platform for producing bioprocess-compatible affinity capture ligands

**DOI:** 10.1016/j.jbc.2023.104910

**Published:** 2023-06-12

**Authors:** Richard J. Suderman, Shane D. Gibson, Mary Strecker, Amanda M. Bonner, David M. Chao

**Affiliations:** 1 Nectagen, Inc, Kansas City, Kansas, USA; 2 University of Washington, Seattle, Washington, USA; 3 Two Dot Consulting, Arvada, Colorado, USA

**Keywords:** affinity chromatography, affinity capture ligand, antibody mimetic, biotechnology, chromatography, protein engineering, protein purification

## Abstract

Protein A affinity chromatography is widely used for the large-scale purification of antibodies because of its high yield, selectivity, and compatibility with NaOH sanitation. A general platform to produce robust affinity capture ligands for proteins beyond antibodies would improve bioprocessing efficiency. We previously developed nanoCLAMPs (nano Clostridial Antibody Mimetic Proteins), a class of antibody mimetic proteins useful as lab-scale affinity capture reagents. This work describes a protein engineering campaign to develop a more robust nanoCLAMP scaffold compatible with harsh bioprocessing conditions. The campaign generated an improved scaffold with dramatically improved resistance to heat, proteases, and NaOH. To isolate additional nanoCLAMPs based on this scaffold, we constructed a randomized library of 1 × 10^10^ clones and isolated binders to several targets. We then performed an in-depth characterization of nanoCLAMPs recognizing yeast SUMO, a fusion partner used for the purification of recombinant proteins. These second-generation nanoCLAMPs typically had a K_d_ of <80 nM, a T_m_ of >70 °C, and a t_1/2_ in 0.1 mg/ml trypsin of >20 h. Affinity chromatography resins bearing these next-generation nanoCLAMPs enabled single-step purifications of SUMO fusions. Bound target proteins could be eluted at neutral or acidic pH. These affinity resins maintained binding capacity and selectivity over 20 purification cycles, each including 10 min of cleaning-in-place with 0.1 M NaOH, and remained functional after exposure to 100% DMF and autoclaving. The improved nanoCLAMP scaffold will enable the development of robust, high-performance affinity chromatography resins against a wide range of protein targets.

Affinity chromatography with target-specific, immobilized capture ligands is an established method of protein purification. In this technique, a capture ligand, such as a protein, nucleic acid, or small molecule, is coupled with a solid support, which can then be used to isolate a protein of interest from a complex mixture. The technique has been widely used at the laboratory scale for single-step purifications of diverse target proteins, including enzymes, transcription factors, growth factors, and antibodies (reviewed in (1)).

The use of protein-based capture ligands for affinity chromatography in process-scale applications has been less widespread because currently available approaches are useful for only a limited number of targets and are incompatible with the temperatures, pH extremes, and solvents often encountered in bioprocessing applications (2). An exception is the purification of kilogram quantities of antibodies with affinity chromatography resins based on *Staphylococcal* Protein A. The development of Protein A resins highlights the use of protein engineering to improve the performance of the capture ligand. Early versions of resins with wild-type Protein A captured antibodies with high selectivity and capacity from crude mixtures but lost activity after multiple cycles of cleaning in place with sodium hydroxide. Mutagenesis of Protein A yielded variants with increased resistance to sodium hydroxide treatment and higher binding capacity (reviewed in (3)). Despite the widespread use of Protein A resins, their use is limited to the purification of antibodies.

For the process-scale purification of non-antibody targets, the use of affinity chromatography is much less widespread than the use of Protein A to purify antibodies. For instance, non-protein, ligand-based approaches, such as small molecule substrate mimetics, are effective but are limited to specific enzyme classes and are difficult to use with a general protein of interest. Alternatively, specialized affinity resins such as glutathione or nickel require the addition of non-native tags, which may cause downstream complications for proteins intended for therapeutic use. Immunoaffinity chromatography with antibody- or nanobody-based capture ligands is the most generally applicable approach and has widely been used to purify a diverse range of proteins at a laboratory scale. However, immunoaffinity chromatography has some limitations. In general, the conjugation of the antibody to the resin often results in heterogenous coupling because of a lack of precise control over the sites of conjugation. In addition, chromatography must be performed under oxidizing conditions in order to preserve the disulfide bonds essential for maintaining antibody structure. Elution of the target also usually requires low or high pH conditions that are incompatible with some target proteins. For process-scale applications, the chief limitation of immunoaffinity chromatography resins is their sensitivity to the sodium hydroxide solutions which are preferred for cleaning-in-place procedures (reviewed in (2)).

These limitations have motivated the development of affinity chromatography resins based on antibody mimetics. Antibody-mimetics are proteins that, like antibodies, can be produced to bind specific antigens with high affinity and specificity but are not directly derived from the immune system of animals. Examples of antibody mimetics include scaffolds based on Protein A, gamma-β crystallin, ubiquitin, cystatin, lipocalins, ankyrin repeat motifs, SH3 domains, fibronectin, OB-fold domains, lamprey variable lymphocyte receptors, minibodies, miniproteins, and Kunitz domains (reviewed in (4)). Most antibody mimetics use animal-sparing phage displays for their isolation and can be produced by microbial cells. Many have a unique cysteine that supports homogeneous, site-specific coupling to sulfhydryl-reactive supports. However, few of the current antibody mimetics have been shown to enable elution near neutral pH or to be compatible with the harsh conditions sometimes needed for process-scale procedures. The development of custom protein-based affinity chromatography platforms for the purification of non-antibody proteins is supported by some proprietary platforms, but the availability and technical details of these platforms are limited (*e.g.*, Avitide, LigaTrap Technologies, Astrea Bioseparations, and Navigo Proteins (reviewed in (5)).

We set out to develop a broadly available and generally applicable platform for isolating protein-based affinity capture reagents compatible with conditions encountered during bioprocessing. Specifically, we sought to develop a platform for the isolation of affinity capture ligands that enable single-step purifications of diverse targets from crude mixtures, allow the elution of target proteins at near neutral pH, and maintain their binding capacity after exposure to high temperatures, organic solvents, proteases, and pH extremes.

We previously developed an antibody-mimetic scaffold based on the 16 kD, second Type 32 carbohydrate binding module of the hyaluronoglucosaminidase nagH from *Clostridium perfringens* (NagH CpCBM32–2) (6). A scaffold is a protein with constant framework regions and variable, antigen-binding regions that are varied to create libraries of binders. This antibody-mimetic scaffold, which produces binding proteins we named nanoCLAMPs (nano Clostridial Antibody Mimetic Proteins), consists of a monomeric β-sandwich domain with variable loops comparable to the complementarity determining regions of the immunoglobulin variable domain. The first-generation nanoCLAMPs have the unusual and advantageous general property of releasing bound target protein in solutions of non-denaturing polyols and ammonium sulfate at neutral pH. We previously isolated nanoCLAMPs recognizing a variety of target proteins with K_d_’s ranging from 10 to 500 nM before affinity maturation (6) and from < 1 PM to 1 nM after affinity maturation (7). These first-generation nanoCLAMPs are defined here as “nC-A” class nanoCLAMPs. Affinity chromatography resins produced with nC-A nanoCLAMPs support single-step purifications to near homogeneity as assessed by Coomassie staining. The working binding capacity of these resins ranges from 5 to 200 nmol target protein per mL of packed beads. While these nC-A nanoCLAMP resins have adequate selectivity and capacity for laboratory-scale purifications, they are incompatible with some bioprocessing conditions because of their moderate thermostability (T_m_ ranging from 45 to 60 °C), sensitivity to proteolytic digestion (t _1/2_ < 1 h in 0.1 mg/ml trypsin), and moderate resistance to alkali (50% loss of activity after 12 cycles of incubation with 0.1 M NaOH).

We set out to improve the performance of the first-generation nC-A nanoCLAMP scaffold to develop a platform for producing custom affinity ligands compatible with bioprocessing conditions. Toward this end, we undertook a multi-round protein engineering campaign to improve the properties of the nanoCLAMP scaffold. In the initial rounds, we made site-directed mutations at specific positions and evaluated the mutations’ impact on thermostability. In later rounds, in addition to analyzing thermostability, we also assessed the mutation’s impact on monodispersity and then combined beneficial mutations to generate the basis for the next round. The end-product of the 7-round, >180 mutation campaign was a clone with significantly improved stability when exposed to heat, proteases, or high pH. We used this clone as the basis for a new nanoCLAMP scaffold, whose variants collectively comprise the “nC-B” class of nanoCLAMPs. Here we report on the campaign to develop the improved nC-B class, the generation and characterization of nC-B nanoCLAMPs against the exemplary protein yeast SUMO (SMT3) and the performance of nC-B nanoCLAMPs after repeated exposure to extreme conditions.

## Results

### Approach to improve scaffold performance with a multi-round campaign of site-directed mutagenesis

We aimed to improve the thermal, proteolytic, and alkaline stability of the first-generation nanoCLAMP scaffold by using consensus protein design, an approach for improving the stability of proteins with many successful precedents (reviewed by (8)). Our initial attempts of directly synthesizing several versions of the consensus sequence were unsuccessful and yielded only aggregated or multimeric proteins. Therefore, we decided to take an incremental approach of making single mutations, determining their effect alone and in combination, and working toward an improved protein in several rounds. We focused the effort on surface residues and loops and generally sought to remove lysine, arginine, and asparagine where possible. The intent of making substitutions for lysine and arginine was to reduce the number of potential sites for cleavage by trypsin. The goal of making substitutions for asparagine was to increase the scaffold’s resistance to the alkaline solutions commonly used to sanitize industrial chromatography columns. Asparagine is often susceptible to deamidation, and its removal has been shown to reduce the loss of protein binding activity in sodium hydroxide (9, 10).

We refer to an individual nanoCLAMP as a “clone,” that is, a specific isolate with unique binding loops as well as regions that remain constant between family members based on the same scaffold. Our starting clone was an anti-SMT3 nanoCLAMP, SMT3-A1 (6), which we previously used as an affinity capture ligand for the laboratory-scale purification of SUMO-fusion proteins in a single step from whole cell extracts. The SUMO-fusion tag is widely used to improve the solubility and yield of proteins produced in *E. coli* and can be cleaved to leave behind a native sequence without any foreign amino acids (11, 12). SMT3-A1 consists of residues 807 to 946 of the second Type 32 carbohydrate binding module of NagH from *C. perfringens*, with variable loops selected for binding to yeast SUMO. Throughout the paper we number nanoCLAMP amino acids based on the sequence of NagH. The variable loops for the nanoCLAMP scaffold are comprised of residues 817 to 820, 838 to 844, and 931 to 935. To identify evolutionarily conserved amino acids, we generated a multiple sequence alignment for a set of 20 non-redundant BLAST hits selected to cover a range of similarity. The percentage identity of the scaffold regions of the orthologs ranged from 56% for *Clostridium nigeriense* to 43% for *Coprobacillus* sp. AF21-8LB (Table S1).

**Figure 1:**
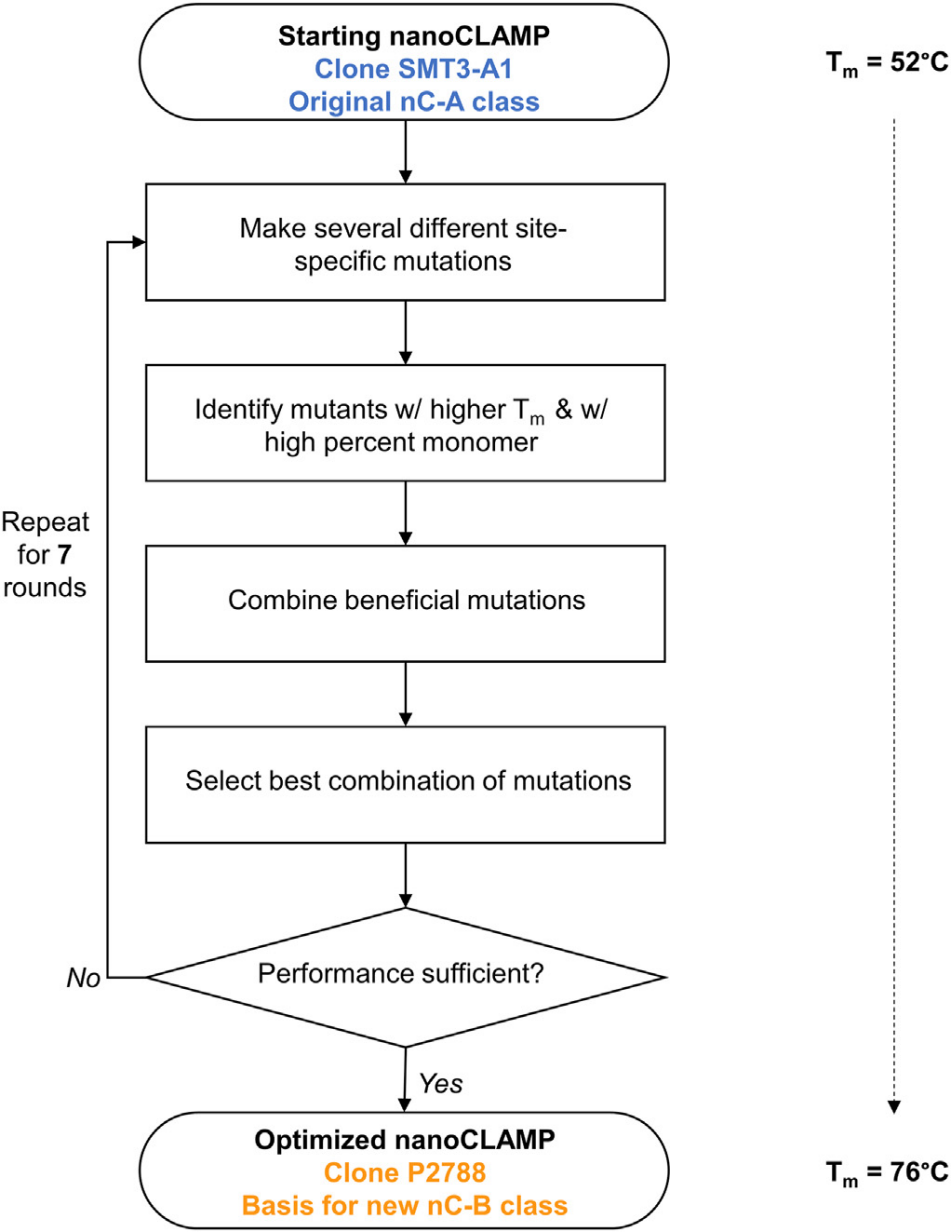
**Outline of protein engineering campaign to produce the nC-B class**. The starting nanoCLAMP was anti-SUMO clone SMT3-A1, a member of the nC-A class of nanoCLAMPs. SMT3A1 was mutated over seven rounds. At the conclusion of each round, the performance of clone(s) combining different mutations from the round was assessed by DSF and SEC. The end product is clone P2788, whose constant regions served as the basis for the nC-B class of nanoCLAMPs.

Our workflow for the protein engineering campaign is summarized in Figure 1. We made site-specific mutations and then purified each individual mutant protein for further biophysical assessment. For the initial assessment, we measured the melting temperature of the resulting mutants using differential scanning fluorimetry (DSF). We then assessed two parameters, melting temperature and initial fluorescence. The rationale for including low initial fluorescence as a criterion for progression is that we observed a rough correlation between high initial fluorescence and the presence of soluble aggregates and multimers detected by size exclusion chromatography. Others have posited that the initial fluorescence in DSF is caused by the binding of fluorophores to hydrophobic patches exposed prior to unfolding (13, 14).

After identifying beneficial mutations, we made several constructs with different combinations of the singly beneficial mutations, whose effects were usually but not always additive. As a secondary screen, we confirmed that the starting clone for each subsequent round of mutagenesis was monodisperse by size exclusion chromatography.

### Optimized scaffold resulting from the mutagenesis campaign

The results of seven rounds of mutagenesis and evaluation are summarized in Table 1. The full list of mutations is listed in File S1.

**Table 1:** Summary of mutation rounds

Round	Clone resulting from round	Total mutations added in round (no.)	Specific mutations added in round	Lysines (no.)	Arginines (no.)	Asparagines (no.)	T_m_	Variants tested
Start	**SMT3-A1**		N/A	10	3	8	52 °C	
1	P2186	8	K857E, E858V, I859V, K860E, L861V, D862G, K870A, K922Q	6	3	8	65 °C	31
2	P2387	5	R812H, R865H, N871D, K897R, K908Q	4	2	7	63 °C	28
3	P2424	7	N832S, K881R, K883R, K901R, E912D, S914D, Q922R	1	6	6	70 °C	33
4	P2550	7	S809T, H812R, S813T, E814P, S815G, D818V N890G, R922Q	1	5	5	71 °C	53
5	P2739	3	R812H, R901H, D818V	1	4	5	74 °C	10
6	P2760	1	W836E	1	4	5	76 °C	2
7	**P2788**	4	N807D, N822S, N825D, N880T	1	4	1	76 °C	27
*Net change*		*30 added*	*N807D, S809T, R812H, S813T, E814P, S815G, D818V, N822S, N825D, N832S, W836E, K857E, E858V, I859V, K860E, L861V, D862G, R865H, K870A, N871D, N880T, K881R, K883R, N890G, K897R, K901H, K908Q, E912D, S914D, K922Q*	*9 removed*	*1 added*	*7 removed*	*+24 °C*	*184 tested*

Bolded font identifies the starting clone.

**Figure 2:**
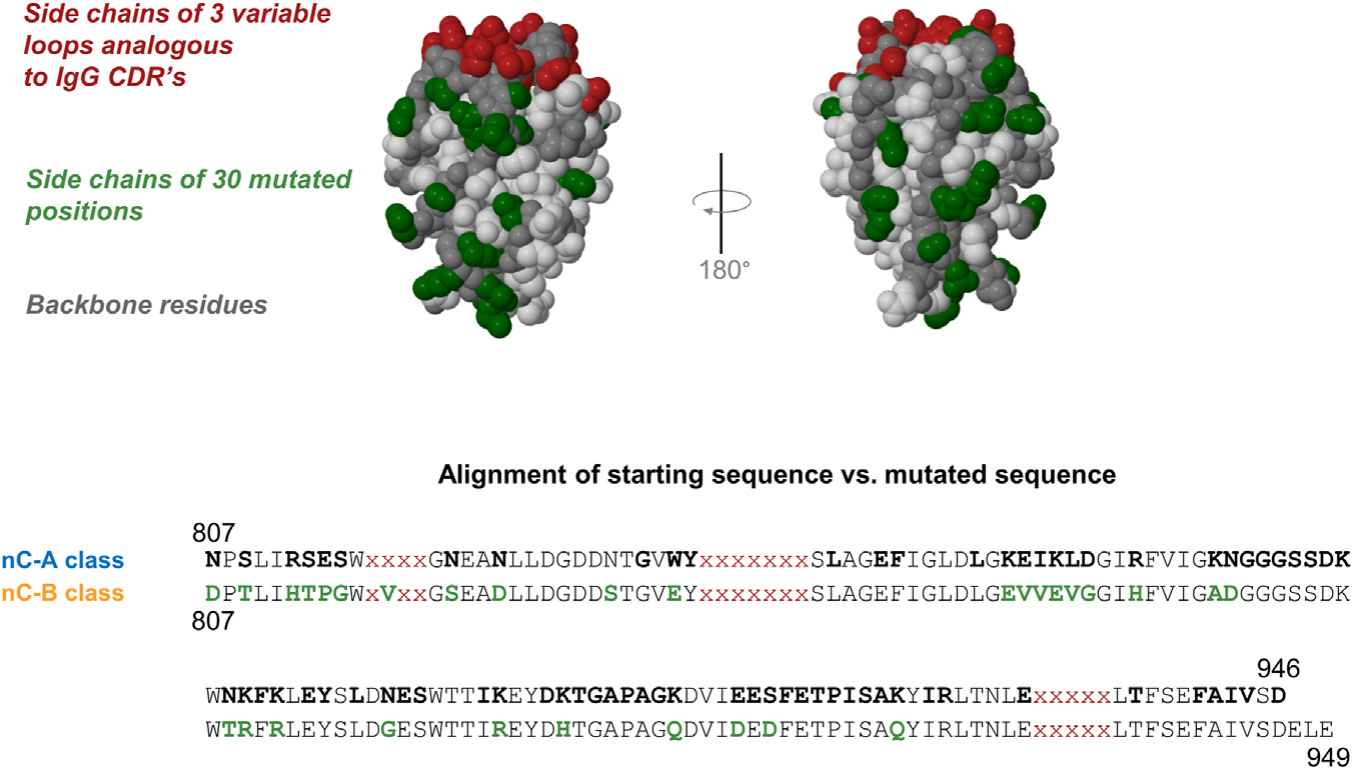
**Mutations in P2788 mapped onto the crystal structure of CBM32-2 and alignment of nC-A and nC-B class nanoCLAMPs**. The constant regions of clone P2788 are the basis for the nC-B class of nanoCLAMPs. Side chains of mutated positions are labeled in *green*. Side chains of variable loops are shown in *red*. Other side chains are shown in *light gray*. Backbone residues are shown in *dark gray*. The alignment compares the constant regions of the nC-A and nC-B classes of nanoCLAMPs. Lower-case x’s denote variable loop residues. Residues denoted with *black, bolded text* represent nC-A positions where at least one mutation was tested (*top row*). Mutations were tested for 58% of positions in the constant regions (72 out of 124). Residues denoted with *bolded text* represent mutations in P2788 (*bottom row*). In P2788, 24% of positions in the constant regions are mutated relative to nC-A (30 out of 124).

Rounds one through four focused on increasing T_m_. Rounds five and six focused on removing potential protease cleavage sites while maintaining T_m_ and included several reversions to past rounds. Round seven focused on removing remaining asparagines while maintaining T_m_. Overall, mutations were tested for 58% of amino acids outside of the variable loops (72 of 124 positions). The clone resulting from seven rounds of mutagenesis is designated P2788. In all, P2788 contains 30 mutations, representing approximately 24% of the positions in the constant regions and includes an additional three C-terminal residues from CBM32-2. The resulting mutations are broadly distributed throughout the primary sequence as shown in an alignment with the original sequence (Figs. 2 and S6) and throughout the 3-D structure when mapped to the CBM32-2 crystal structure (PDB accession 2W1Q) (15). Although the mutants all possessed the same binding loops as the initial clone nanoCLAMP SMT3-A1, we expected and observed a gradual decline in target binding with an increasing number of mutations, many of which were adjacent to the binding loops. We speculate that the loss of binding was caused by shifts in the conformation of the binding loops (data not shown). Because our intent was to improve the stability of the constant regions of the scaffold and then isolate new binders, our screening funnel did not include an assessment of SUMO binding.

The number of lysines and arginines representing potential trypsin cleavage sites was reduced from 11 in the scaffold of the starting protein (clone SMT3-A1) to five in the scaffold of the resulting protein (clone P2788). Three of the remaining arginines (R881, R897, and R925) are expected to be involved in salt bridges as identified by the ESBRI algorithm (16). For these residues, we were unable to identify any substitution mutations that did not destabilize the proteins. For another position, K883, substitution with arginine was beneficial, but several additional substitutions either resulted in a >10 °C decrease in melting temperature or high initial fluorescence in DSF. For one remaining position, K878, which is universally conserved in the alignment, 10 of 10 substitution mutations resulted in proteins with high initial fluorescence in DSF. In the wild-type NagH CpCBM32-2 structure, the K878 Nζ forms hydrogen bonds with the carbonyl oxygens of P905 and G907 as determined by the RING 2.0 algorithm (17). The loss of K878 side chain interactions may cause substitution mutations to destabilize the protein. The number of asparagines was reduced from eight in the original scaffold (SMT3-A1) to one in the mutated scaffold. The remaining asparagine N928 is universally conserved in the consensus alignment and buried in the 3-D structure. N928 Nδ^2^ forms hydrogen bonds with the carbonyl oxygens of S845 and L846 as determined by the RING 2.0 algorithm. We chose not to attempt substitution mutations with N928 because of the low likelihood of deamidation based on its sequence context and the likely challenge of finding a substitution with a beneficial effect.

**Figure 3:**
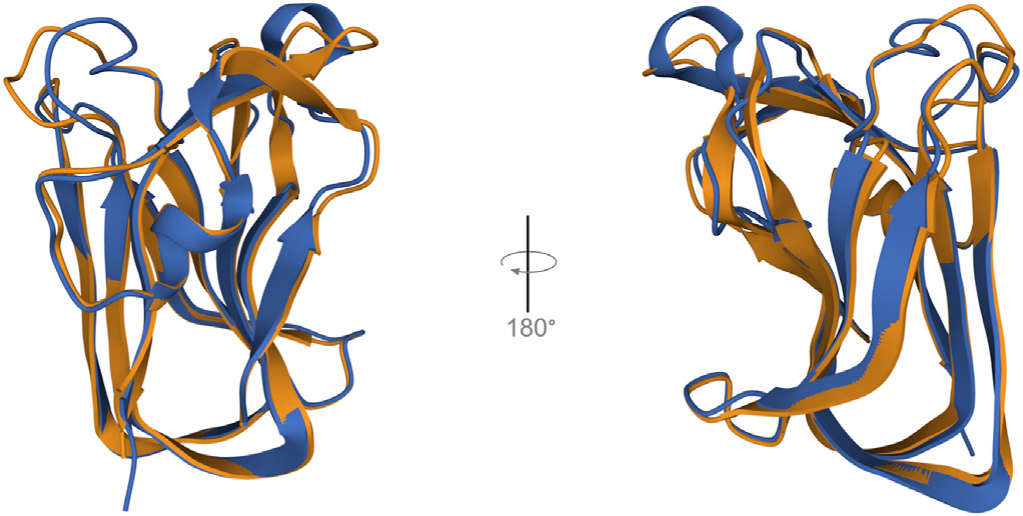
**Superimposition of crystal structure for CBM32-2 (basis for nC-A class) and AlphaFold prediction for P2788 (basis for nC-B class)**. CBM32-2 (PDB accession 2W1Q), shown in *blue*, and P2788, shown in *gold*, were aligned with jFATCAT (*rigid*) on the RCSB server. Backbone deviations are apparent for the loops, which have different amino acid sequences. The TM-score is high (0.95).

We next used AlphaFold to predict the 3-D structure of P2788 in order to assess the likelihood of gross changes in 3D-structure (18). A 3-D alignment of the crystal structure of CBM32-2 and the predicted structure of P2788 was performed with the jFATCAT(rigid) algorithm (Fig. 3). As expected with conservative substitutions, the high degree of similarity and the use of templates by the AlphaFold algorithm, the predicted structure of the constant regions of P2788 does not show gross deviations from the solved crystal structure of NagH CpCBM32-2. Because of differences in the amino acid sequences of the loops, we expected and observed the variable loops to show deviations in predicted structure, especially for the longest 838 to 844 loop. The overall similarity of the solved CBM32-2 structure *versus* the predicted structure of P2788 is high, even with the differences in loop sequences (TM-score = 0.95).

**Figure 4:**
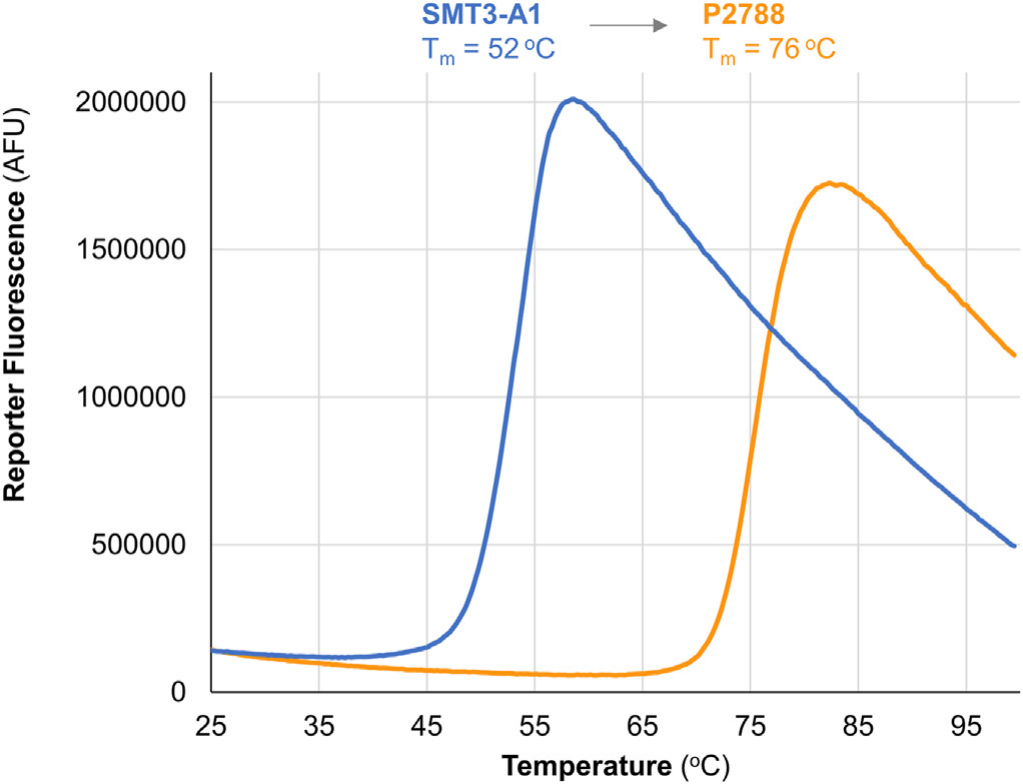
**Differential scanning fluorimetry analysis of SMT3-A1 (nC-A class) and P2788 (nC-B class)**. Both clones show classically shaped melting curves with low initial fluorescence. The 30 mutations in P2788 increase its T_m_ by 24 °C relative to SMT3-A1.

**Figure 5:**
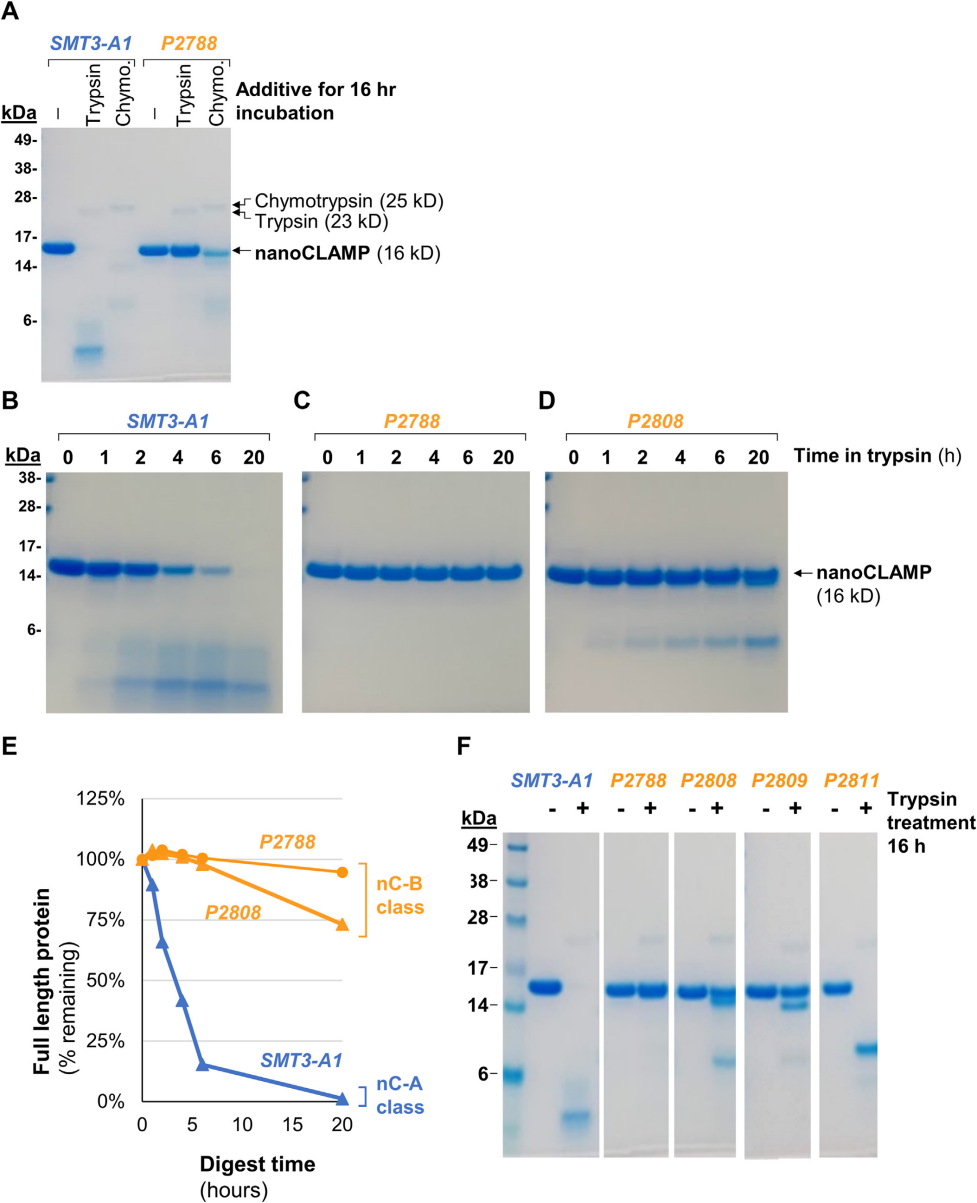
**Protease resistance of nC-A and nC-B class nanoCLAMPs**. *A*, SDS-PAGE analysis of SMT3-A1 (nC-A class) and P2788 (same variable loops as SMT3-A1 but with constant regions of the nC-B class) after a 16 h incubation with trypsin or chymotrypsin. *B–D*, SDS-PAGE analysis of time course tryptic digestions of SMT3-A1, P2788 and P2808. P2788 and P2808 have the same constant regions (nC-B class) but different loops. P2788 and P2808 were resistant to tryptic digestion for over 20 h. *E*, quantitative densitometry analysis of the stained gels containing samples from the time-course experiment. *F*, SDS-PAGE analysis of members of the nC-A class of nanoCLAMPs (SMT3-A1) and nC-B class (P2788, P2808, P2809, and P2811) following 16 h tryptic digestions. *White borders* indicate lanes rearranged from the same gel or different gels (processed identically).

Compared with the starting protein (SMT3-A1), the T_m_ of P2788 increased by 24 °C from 52 °C to 76 °C. (Fig. 4). We next tested P2788’s resistance to digestion by trypsin. Following a 16 h digestion in 0.1 mg/ml trypsin, no full length SMT3-A1 remained as assessed by SDS-PAGE. In contrast, no apparent digestion of P2788 had occurred (Fig. 5*A*). A time course of trypsin digestion determined that the t_1/2_ increased from 3 h for SMT3-A1 to > 16 h for P2788 (Fig. 5, *B*, *C* and *E*). While our protein engineering campaign deleted only a few surface-exposed chymotrypsin-cleavage sites, we also tested resistance to chymotrypsin as a reflection of general stability. Following a 16 h digestion with 0.1 mg/ml chymotrypsin, about a fifth of P2788 appeared to remain full-length while SMT3-A1 was completely digested (Fig. 5*A*).

We next sought to determine whether a scaffold based on the constant regions of P2788 could confer these properties to newly isolated clones. We call the P2788-derived class the “nC-B class” (nanoCLAMP-B with the identifier “B” referring to the next-generation class of nanoCLAMPs). Throughout the figures, clones of nanoCLAMPs of the nC-B class are labeled in orange. The first generation of nanoCLAMPs represented by SMT3-A1 and others is referred to as the “nC-A class” (nanoCLAMP-A with the identifier “A” referring to the first-generation class of nanoCLAMPs). For clarity, the nC-A class of nanoCLAMPs encompasses the first published nanoCLAMPs (6). Relative to NagH CpCBM32-2, nanoCLAMPs of the nC-A class have a M929L mutation that removes a methionine as well as amino acid differences in the variable loops. Clones of nanoCLAMPs of the nC-A class are labeled in blue throughout the figures.

### Phage display library panning for improved SUMO-binding nanoCLAMPs based on the nC-B scaffold

To confirm that the optimized constant regions of the nC-B class scaffold can support the general isolation of high-affinity binders with improved tolerance to heat, proteases, and high pH, we constructed a phage display library with randomized binding loops in the context of the nC-B constant regions and panned the library for binders to yeast SUMO (SMT3). This library has the same three variable loop positions as the previous library from which SMT3-A1 was isolated but uses the nC-B scaffold instead of the nC-A scaffold. Degenerate oligonucleotides constructed with phosphoramidite trimers were designed so that the variable regions encoded all amino acids except cysteine (omitted to avoid heterogeneous coupling to multiple cysteines), methionine (omitted to avoid the risk of inactivating oxidation), and lysine and arginine (omitted to avoid the addition of trypsin-cleavage sites). Position 818 was held constant with a valine, the wild-type amino acid, because valine and another small hydrophobic amino acid isoleucine, appeared in one-quarter of nanoCLAMPs from previous screens. The resulting library contained over 10^10^ variants of nC-B nanoCLAMPs.

Following the third round of panning of this library, we randomly selected 96 clones and screened them for target binding by semELISA, which yielded 93 confirmed positives. Of these, 40 were sequenced to identify 18 unique nanoCLAMP SUMO binders. These binders were subcloned into a bacterial expression vector, expressed, purified by IMAC, and confirmed to be over 90% pure as estimated by SDS-PAGE (data not shown). We then evaluated the purified nanoCLAMPs’ ability to function as affinity capture ligands in a medium-throughput, small-scale depletion assay. In this assay, the nanoCLAMPs were conjugated to cross-linked agarose under denaturing conditions, refolded on the resin, incubated with SUMO, and the quantity of unbound SUMO was measured by A_280_.

The purified nanoCLAMPs were then screened for monodispersity by size exclusion chromatography and T_m_ by DSF. Of the 18, seven (38%) were over 90% monomer. Of these, five had a melting temperature of greater than 73 °C, with four having melting temperatures greater than 99 °C (Table S2). The initial results with the SUMO test case suggest that the nC-B constant regions generally support the isolation of clones with high monodispersity and thermostability. To determine whether the high thermal stability and monodispersity observed for the SUMO binders was a general property of nanoCLAMPs isolated with this scaffold, we panned for and characterized binders to two additional protein targets. The median T_m_ of 22 positive binders (identified by ELISA) to Target one was 78 °C and 45% of the binders were over 75% monodisperse monomer. The median T_m_ of the 39 binders to Target two was 71 °C, and 62% were over 75% monodisperse monomer (Table S3). These results align well with the SUMO binder data and support the utility of the new scaffold for identifying thermostable, monodisperse binders to different targets.

### Characterization of binding affinity, thermostability, and protease-resistance of capture ligands based on the nC-B scaffold

We chose three nC-B nanoCLAMPs (P2808, P2809, and P2811) with diverse binding loops for further characterization. For the three clones, Loop 817 to 820 and Loop 931 to 935 did not have any apparent similarity. Except for V818, whose identity was fixed in the library, there were no identities in any positions of these loops. For Loop 838 to 844, clones P2808 and P2809 are identical in five of seven positions while clone 2811 shows no identities with either. All three nanoCLAMPs were produced in *E. coli* shake flasks with yields > 150 mg/L of culture and used for biophysical characterization experiments.

**Figure 6:**
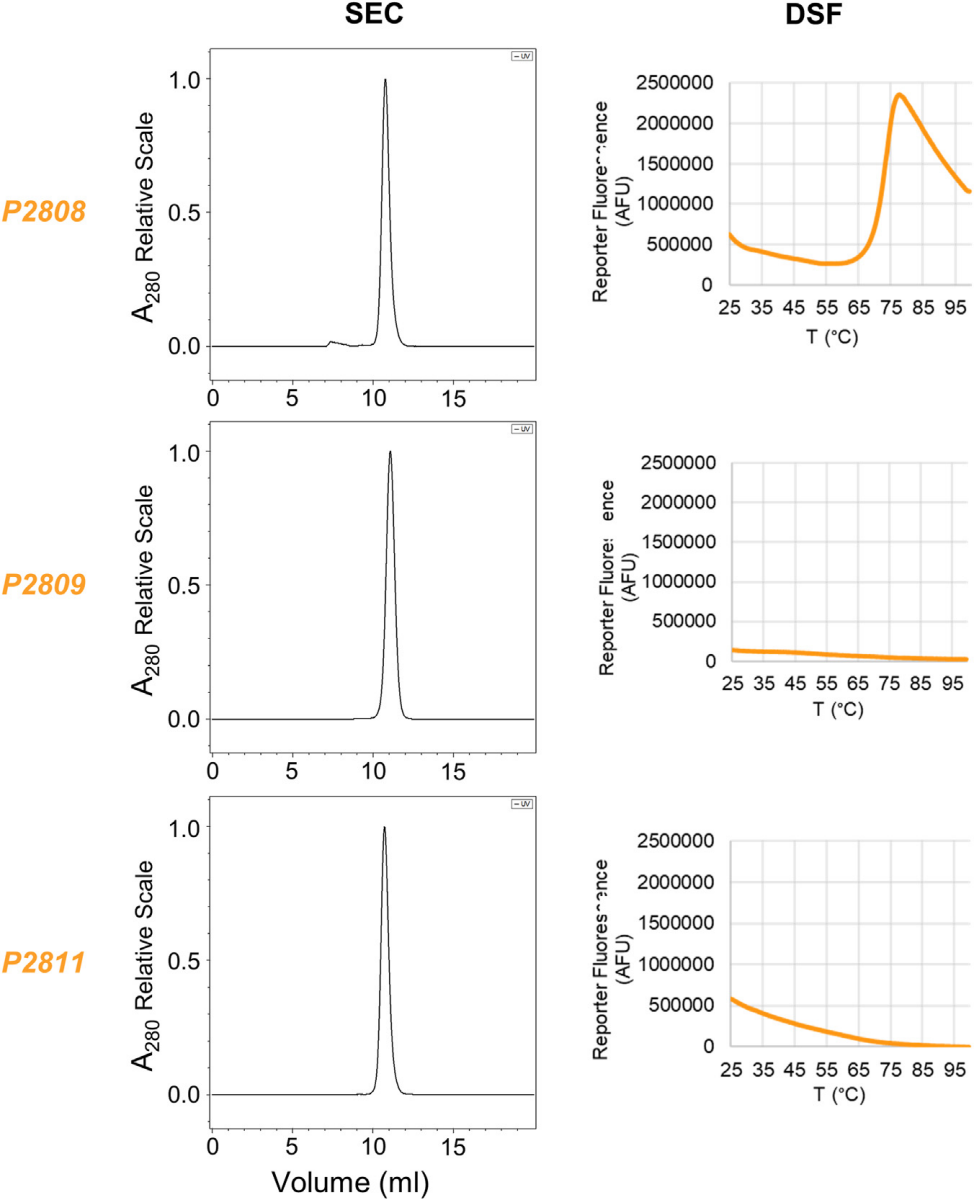
**Monodispersity and melting temperature analysis of anti-SUMO nanoCLAMPs of the nC-B class**. Size exclusion chromatography (*left panel*) and differential scanning fluorescence (*right panel*) of nanoCLAMPs P2808, P2809 and P2811.

We first checked the quaternary structure of nanoCLAMPs by size exclusion chromatography to confirm that subsequent results could be interpreted without confounding avidity effects from higher order multimers or aggregates. All three clones eluted as monodisperse monomers (Fig. 6). To rank these nanoCLAMPs by their affinity for SUMO, we used biolayer interferometry to measure their dissociation constants, which ranged from 5 to 80 nM (Table 2 and Fig. S7). We then measured the melting temperature of the nanoCLAMPs using DSF. P2808 had an apparent T_m_ of 73 °C. P2809 and P2811 had flat-line DSF curves with no melting transition apparent between 25° and 99 °C (Fig. 6). This observation suggests that the T_m_ for these nanoCLAMPs exceeds the quantifiable range for this assay. We corroborated this observation with a functional binding assay to assess kinetic thermostability. In this assay, we incubated samples of each nanoCLAMP at different temperatures, cooled and centrifuged the solutions, and then measured the binding activity remaining in the supernatant by biolayer interferometry. In this test, we define T_50_ as the temperature of a 5 min heat challenge after which 50% of binding activity is irreversibly lost (reviewed in (19)). The T_50_ ranged from ∼85 °C for clone P2808 to > 100 °C for clones P2809 and P2811. The rank order and values are consistent with the T_m_ measured by DSF (Fig. S1). Clones P2809 and P2811, both with T_m_’s > 100 °C, maintained greater than 90% activity after incubation at boiling temperatures for 5 min. The maintenance of binding activity indicates that the nanoCLAMPs remained in solution after heat treatment and did not irreversibly aggregate or precipitate. Taken together with the DSF data, the kinetic thermostability measurements suggest that these two nanoCLAMPs may remain folded up to, and possibly above, 99 °C.

**Table 2:** T_m_, T_50_, K_d_, trypsin resistance of selected anti-SUMO nanoCLAMPs

Clone	nanoCLAMP class	Loop seq. 817–820	Loop seq. 838–844	Loop seq. 931–935	T_m_	T_50_	K_d_ SUMO	Trypsin resist.
SMT3-A1	nC-A	EDIK	FNEVFYE	DKILF	52 °C	∼58 °C	232 nM	−
P2788	nC-B	EDIK	FNEVFYE	DKILF	∼76 °C	N/A	N/A	+
P2808	nC-B	LVIP	IIVEEFT	YNFID	73 °C	∼85 °C	7 nM	+
P2809	nC-B	NVEQ	ILVEEFP	NEVPQ	>99 °C	>100 °C	80 nM	+
P2811	nC-B	PVVT	IVLTDQA	LAQDP	>99 °C	>100 °C	5 nM	−

We next characterized the trypsin resistance of the three clones. Clones P2808 and P2809 were highly resistant to digestion with 0.1 mg/ml trypsin while clone P2811 was less resistant (Fig. 5*F*). Figure 5, *B*–*D* shows a time course of a tryptic digest comparing nanoCLAMPs with different combinations of constant regions and loops to understand the contribution of each component to trypsin resistance. We tested the original clone SMT3-A1 (nC-A constant regions and original variable loops), P2788 (nC-B constant regions with the original variable loops from SMT3-A1), and P2808 (nC-B constant regions with newly isolated variable loops). Both P2788 and P2808 show a t_1/2_ of > 20 h in 0.1 mg/ml trypsin, compared with ∼4 h for the original SMT3-A1 clone. Together with the observations made with clones P2809 and P2811, these results indicate that trypsin-resistance depends upon the sequences of both the variable loops and the constant regions. The observation of trypsin resistance for three of four clones isolated with diverse loop sequences and identical constant regions indicates that the nC-B constant regions can be generally used to isolate trypsin-resistant clones in at least a first test-case.

### Measurement of performance parameters with affinity chromatography using capture ligands based on the nC-B scaffold

For brevity, we will subsequently refer to affinity chromatography resins made with affinity capture ligands of the nC-B class of nanoCLAMPs as “nC-B affinity resins.” As a test case for the utility of nC-B affinity resins, we used P2808 as a capture ligand for more detailed studies. To generate the affinity resin, the P2808 protein was expressed, purified by IMAC under denaturing conditions, conjugated to sulfhydryl-reactive 6% cross-linked agarose resin, and then refolded by rinsing with buffered saline. We prepared a test mixture of crude *E. coli* lysates spiked with a SUMO-GFP fusion protein, optimized purification conditions, and then assessed the resins’ selectivity and binding capacity.

In pilot experiments, we observed that polyol-elution at neutral pH was still possible, as with the original nanoCLAMP (6), but with qualitatively lower speed and yield (data not shown). As an alternative, we tested the elution of the target protein with imidazole, which has been used successfully for disrupting protein-peptide interactions (20) and antibody-Protein A interactions (21).

A buffer with 3 M imidazole at pH eight quickly and completely eluted SUMO from P2808 resin. As shown in Fig. S2, after incubation for 1 h with an excess of target protein in a spiked *E. coli* lysate, the bound target was washed and eluted with a purity of over 90% as estimated by densitometric analysis of Coomassie stained SDS-PAGE. The static binding capacity under these conditions was 11.5 mg/ml resin (277 nmol/ml). In subsequent experiments, we found that imidazole elution was similarly effective with P2808, P2809, and P2811, three additional nC-B class binders to different targets (Fig. S5), as well as three different resins targeting SUMO, mCherry, and GFP with capture ligands of the nC-A class (data not shown). The observations suggest that imidazole elution is a general property of the nanoCLAMPs.

**Figure 7:**
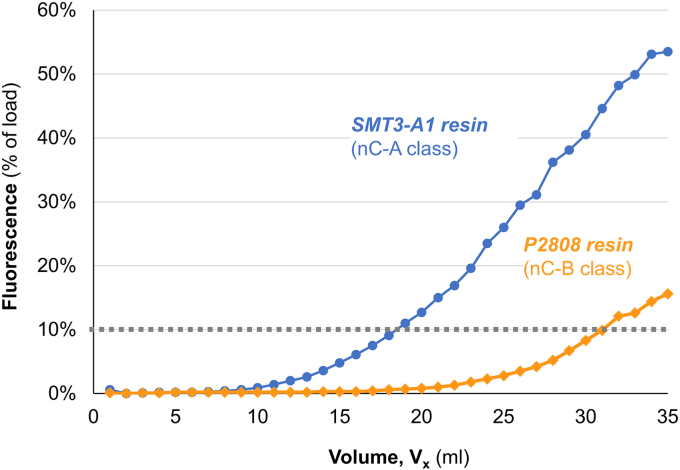
**Dynamic binding capacity of SMT3-A1 resin (nC-A class) and P2808 resin (nC-B class)**. Breakthrough curves were generated by loading a solution of 0.2 mg/ml SUMO-GFP in PBS onto 0.6 ml of packed resin in a column (3 cm height × 5 mm ID) at a flowrate of 0.5 ml/min and then measuring the fluorescence of the eluate. The percentage of fluorescence of the load was calculated by dividing the eluate fluorescence by the load fluorescence. The dynamic binding capacity (DBC) was calculated with the following formula: DBC = (V_x_–V_delay_) ∗c/(V_resin_). V_x_ is the volume of eluate collected; V_delay_ is the elution volume of the load under non-binding conditions, c = concentration of target in load; V_resin_ is the volume of the packed resin in the column. The P2808 resin has a dynamic binding capacity of 10 mg/ml resin (240 nmol/ml resin).

With elution conditions established, we next measured the dynamic binding capacity of P2808 resin using a 0.6 ml packed column (0.5 cm ID × 3.06 cm) at a constant flowrate of 0.5 ml/min. We utilized the fluorescence of the SUMO-GFP target to determine the QB_10_ (the volume at which the eluate’s fluorescence equaled 10% of the load’s fluorescence; see Materials and Methods for calculations). The dynamic binding capacity of P2808 resin under these conditions was 10 mg/ml resin (240 nmol/ml resin) (Fig. 7). This capacity represents an approximate 70% increase over our original SMT3-A1 resin.

We next tested the efficiency of the SUMO affinity resin P2808 resin under flow conditions. A 0.6 ml packed column and a flowrate of 0.5 ml/min (linear flowrate = 153 cm/h) were used to test two regimes.

**Figure 8:**
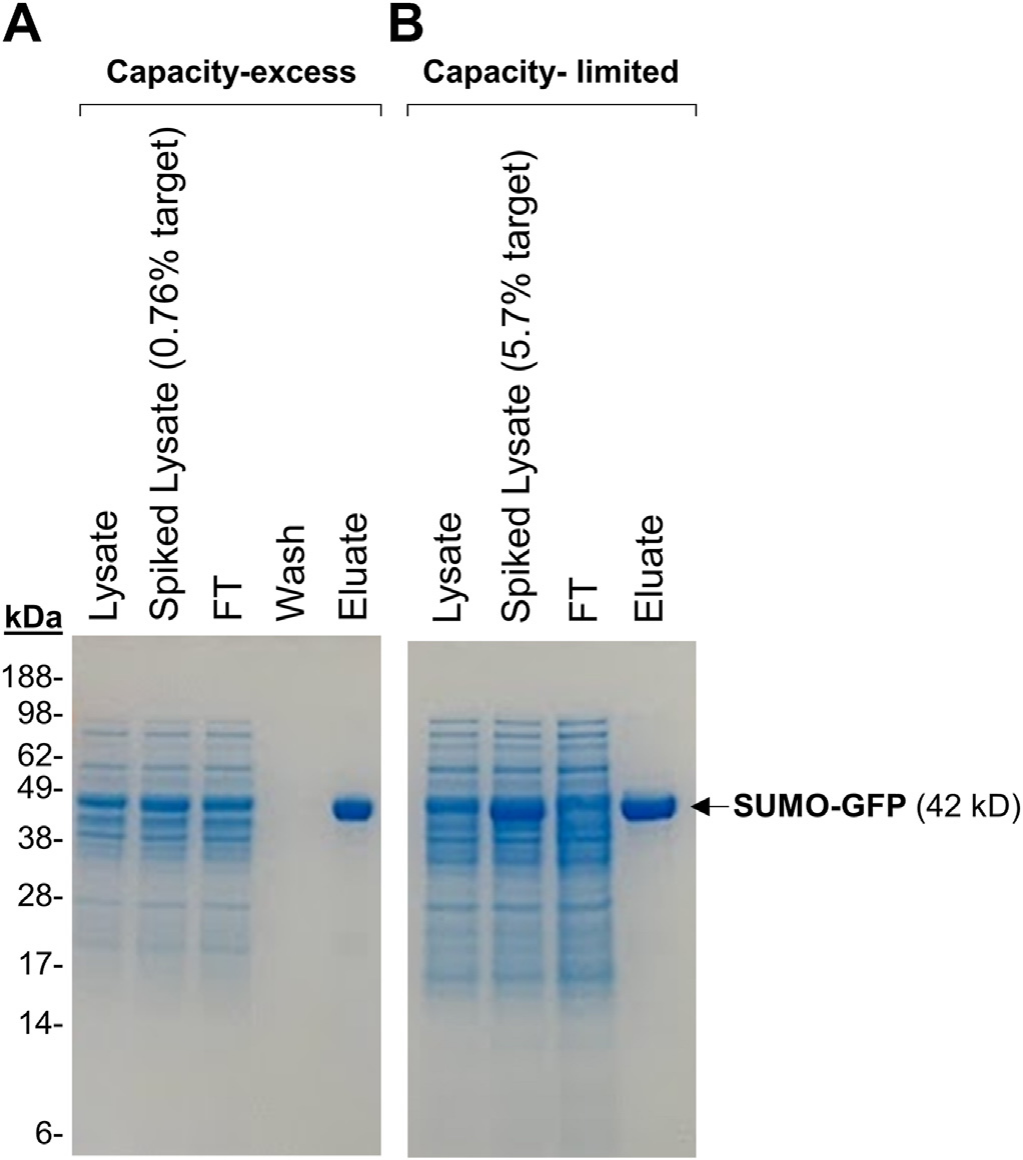
**Performance of P2808 resin in single-step affinity chromatography purification of GFP-SUMO from spiked lysates in resin-limiting and protein-limiting scenarios**. Identical columns were loaded under conditions that were 33% above (*A*) or 58% below (*B*) the column’s dynamic binding capacity. *E. coli* lysates with spiked-in target protein (SUMO-GFP) were used as the load. After loading and washing, bound proteins were eluted with 3 M imidazole pH 8. Total protein loaded on SDS-PAGE in *Panel A*: Lysate = 32 ug, Spiked Lysate = 34 μg, FT = 47 μg, Eluate = 6 μg; *Panel B*: Lysate = 17 μg, Spiked Lysate = 17 μg, FT = 21 μg, Wash = N/A, Eluate = 3 μg. Metrics of purifications *A* and *B* are tabulated in Table 3.

In the capacity-excess regime, SUMO was spiked into an *E. coli* lysate at a low concentration (SUMO-GFP = 0.76% of total protein by weight), loaded at 42% of the column’s dynamic capacity (36% of its static binding capacity). As assessed by densitometry of a Coomassie-stained SDS-PAGE gel, the purity was > 90%, and the yield was 90%. (Fig. 8*A*).

In the capacity-limited regime, SUMO-GFP was spiked into an *E. coli* lysate at a high concentration (SUMO-GFP = 5.7% of total protein by weight) and loaded at 133% of the column’s dynamic binding capacity (116% of its static binding capacity) (Fig. 8*B*). The purity and yield were comparable to the capacity-excess regime and estimated at > 90% and 90%, respectively. The performance metrics for both purifications are summarized in Table 3.

**Table 3:** Metrics for AC purification of SUMO-GFP with P2808 resin loaded at different capacities

Metric	Capacity-excess	Capacity-limited
% of dynamic binding capacity^a^	42%	133%
% of static binding capacity^b^	36%	116%
Total protein in Load (mix of *E. coli* lysate and SUMO)	333 mg	140 mg
Target (SUMO-GFP) in Load	2.5 mg (0.76% of total protein)	8 mg (5.7% of total protein)
Concentration of target in Load	0.025 mg/ml	0.2 mg/ml
Volume of Load	100 ml	40 ml
Flow rate	0.5 ml/min (0.44 CV/min)	0.5 ml/min. (0.44 CV/min)
Linear flow rate	153 cm/h	153 cm/h
Residence time	1.2 min	1.2 min
Recovered target	2.47 mg	7.6 mg
Purity by densitometry	>90%	>90%
Fold purification	120-fold	16-fold
Yield	>90%	>90%

a DBC = 10 mg/ml resin (see Fig. 7).

b SBC = 11.5 mg/ml resin (see Fig. S2).

Finally, to make sure that P2808 resin would function similarly with SUMO fusions other than SUMO-GFP, we tested the resin in a small-scale batch affinity chromatography purification using SUMO-NusA as a target. As expected, SUMO-NusA was purified to greater than 95% purity (as determined by SDS-PAGE) in a single step from a spiked *E. coli* lysate using 3 M imidazole as eluant, suggesting the resin is accommodating to diverse SUMO-fusions (Fig. S5, lanes 1, 2).

### Compatibility of nC-B affinity resins with repeated cycles of NaOH cleaning

**Figure 9:**
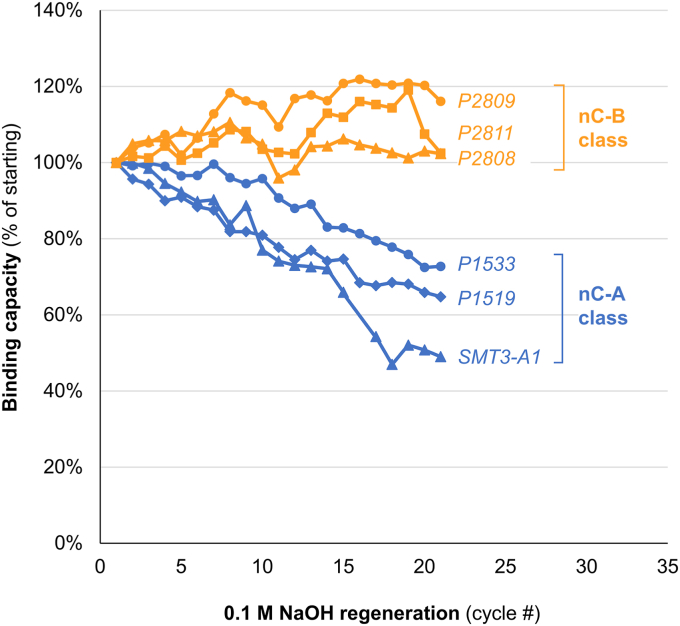
**Effect of sodium hydroxide treatment on binding capacity of nanoCLAMP affinity capture ligands based of the nC-A and nC-B class**. The binding capacities of resins with capture ligands based on the nC-A class (SMT3-A1, P1519, P1533) and the nC-B class (P2808, P2809, P2811) were determined after each of 22 cycles of purification of GFP-SUMO from a spiked *E. coli* lysate, followed by washing, eluting, and cleaning in place with 0.1 M NaOH (10 min contact time). The percentage of starting binding capacity was determined by dividing the eluate fluorescence with the load fluorescence. The selectivity was determined by analysis of the eluates on SDS-PAGE (Fig. S3).

For large-scale production of industrial proteins or biologics, the re-use and cleaning of columns in place reduces manufacturing costs and maintains consistent performance. Sodium hydroxide solutions are commonly used for cleaning-in-place procedures (22) so we tested the general compatibility of the new nanoCLAMP resins with sodium hydroxide treatment. We tested three resins made with nC-A nanoCLAMPs and three resins made with nC-B nanoCLAMPs. Each cycle consisted of loading an *E. coli* lysate spiked with SUMO-GFP; a wash step; elution with 3 M imidazole pH 8; 10 min of contact time with 0.1 M NaOH; and then a 5 min re-equilibration step. The eluates were collected and analyzed for purity by SDS PAGE, and the purified target was quantified by fluorescence spectroscopy (Figs. 9 and S3). In the first few cycles, we were surprised to observe an improvement in binding capacity of 5 to 20% with nC-B resins. The increase in binding may result from the removal of inhibitory material by NaOH, but we have not investigated this phenomenon further. For all three nC-B resins, the binding capacity plateaued over 20 cycles and remained at or above 100% of starting capacity. In contrast, we observed a steady reduction in binding capacity by 25% to 50% with all three nC-A resins. Taken together, these data indicate that nanoCLAMPs of the nC-B class can generally serve as capture ligands for resins capable of single-step affinity purification of targets to homogeneity. These resins are also generally compatible with cleaning-in-place protocols using 0.1 M sodium hydroxide for over 20 cycles, without loss of binding capacity or specificity. Further, in practice, cleaning-in-place cycles are not usually performed after each run, so the expected lifetime of nanoCLAMP resins likely exceeds 100 cycles with the assumption of sanitation every fifth run.

### Compatibility of nC-B affinity resins with repeated cycles of low pH elution

Because elution with 3 M imidazole might be suboptimal for some applications, we also tested elution with a citrate buffer at pH 2.5. In these experiments, the resin was also cleaned with NaOH in between each cycle for 1 min before re-equilibrating the resin with buffered saline. The nanoCLAMP maintained 100% of its binding capacity and specificity over more than 20 cycles of loading, elution, and regeneration (Fig. S4).

### Compatibility of nC-B affinity resins with organic solvent and autoclaving

**Figure 10:**
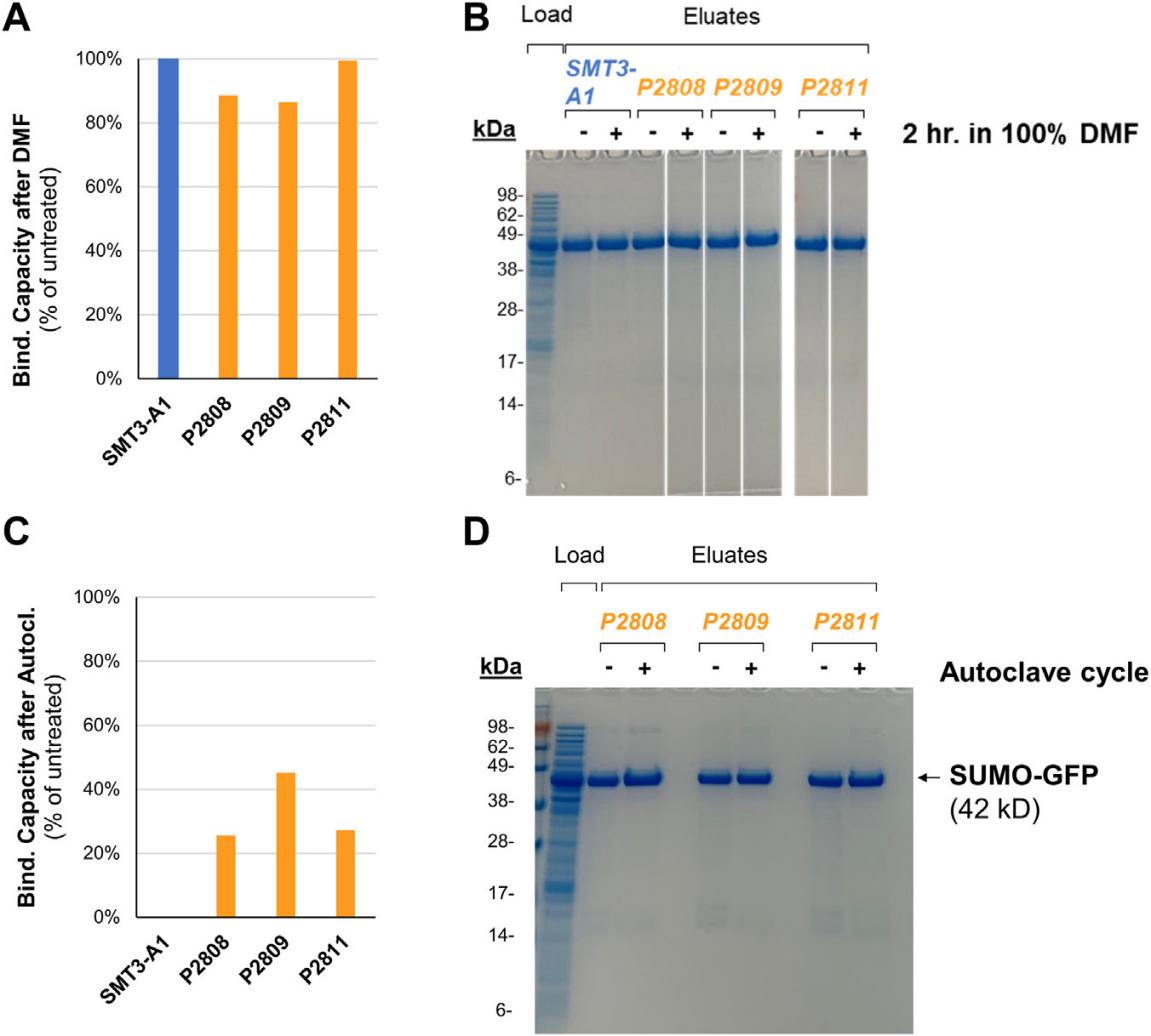
**Effect of organic solvent and autoclaving on the binding capacity of nanoCLAMP resins made with nanoCLAMPs of the nC-B class**. Resins P2808, P2809, and P2811 (nC-B class) and Resin SMT3-A1 (nC-A class) were incubated in 100% DMF for 2 h (*A* and *B*) or autoclaved (105-min liquid steam cycle, including 30 min exposure to 120 °C and 20 p.s.i.) (*C* and *D*) and then re-equilibrated in fresh buffer and tested in affinity chromatography purification of SUMO-GFP from spiked *E. coli* lysate. The binding capacity of treated resin as a percentage of untreated resin was determined by dividing the eluate fluorescence by the control (non-treated) eluate fluorescence. Specificity was assessed by Coomassie staining of SDS-PAGE gels (Fig. 10, *B* and *D*). *White borders* indicate lanes rearranged from the same gel or different gels (processed identically).

We next tested the ability of the nC-B resins and the original SMT3-A1 resin to resist more extreme conditions. First, we tested the resins’ ability to recover selective binding capacity after exposure to 100% DMF. We incubated nanoCLAMP resin in 100% DMF for 2 h, re-equilibrated with buffered saline, and then measured binding capacity. Of the four resins tested (the original SMT3-A1 resin and P2808 resin, P2809 resin, and P2811 resin), all retained over 85% binding capacity after treatment with DMF (Fig. 10*A*) and maintained their apparent selectivity as assessed qualitatively by SDS-PAGE (Fig. 10*B*).

Because the nC-B resins were robust to the broad range of conditions tested so far, we decided to determine whether the resins could also retain binding and specificity after autoclaving. We autoclaved the resin with a 105 min liquid steam cycle, including 30 min exposure to 120 °C and 20 p.s.i., re-equilibrated at room temperature, and then measured binding capacity. The resin made with the nC-A nanoCLAMP (SMT3-A1) did not bind any detectable target protein after autoclaving. In contrast, the resins made with the nC-B nanoCLAMPs (P2808, P2809, and P2811) retained 25% to 45% binding capacity, with specificity comparable to controls (Fig. S10, *C* and *D*). The SMT3-A1 eluates were not analyzed in Figure 10*D* because there was not enough protein in the autoclaved sample to prepare a normalized aliquot to compare to the control. To our knowledge, affinity chromatography resins made with second-generation nanoCLAMPs are the first protein-based affinity resins shown to retain significant binding capacity and specificity after autoclaving.

### Demonstration of wide applicability of nC-B scaffold for identifying high-performance affinity ligands to diverse targets

Finally, we tested whether we could use the new nC-B scaffold to generate affinity resins to diverse targets. We panned the NL-26 phage display library against SARS CoV 2 receptor binding domain (RBD) (alternating between the Wuhan and Delta variants each round), the extracellular domain (ECD) of human CTLA-4 (Cytotoxic T-Lymphocyte Antigen 4, a protein receptor that downregulates the immune system) and the ECD of human TIGIT (a transmembrane protein expressed at low levels on peripheral memory and regulatory CD4+ T-cells and NK cells that is up-regulated following activation). These recombinant, glycosylated protein targets were purified from HEK293 cells, whereas the targets described above were non-glycosylated proteins purified from *E. coli*. Following the same panning and characterization process described above, we selected lead candidates P3114 (RBD binder), P2874 (CTLA-4 binder), and P2978 (TIGIT binder) for incorporation into affinity resins due to their high thermal stability and monodispersity (Table S2). We spiked RBD into expi293 supernatant and CTLA-4 and TIGIT into *E. coli* crude lysates and tested these affinity resins’ performance in small scale batch affinity chromatography. As shown in Fig. S5, lanes 3 to 8, each target was purified to greater than 95% purity in a single step, as determined by SDS-PAGE analysis of eluates, and all three resins were fully elutable using 3 M Imidazole, pH 8, suggesting that the nC-B scaffold should be able to produce high affinity, high specificity affinity ligands for the purification of a diverse array of targets.

## Discussion

We report on a protein engineering campaign yielding an improved class of nanoCLAMPs with utility as capture ligands for downstream bioprocess affinity chromatography. The mutations which we selected to the original class A nanoCLAMPs resulted in significantly improved (24 °C) thermal stability and improved overall monodispersity of clones with varying loops. We speculate that these mutations improved hydrophobic interactions in the protein core leading to a tighter, more thermally stable core with decreased hydrophobic exposure to the aqueous environment, decreasing intermolecular hydrophobic interactions; however, this will need to be tested by a detailed structural analysis. Resins made with the new nC-B class of nanoCLAMPs support the high-yield capture of target proteins from complex mixtures, similar to Protein A resins and antibody-based immunoaffinity resins. Similar to Protein A resins, but unlike antibody-based resins, nC-B resins are compatible with NaOH cleaning-in-place, use capture ligands that lack cysteines, and can be produced by bacterial expression. Unlike either Protein A or antibody-based resins, nC-B resins are distinct in exhibiting resistance to boiling temperatures, trypsin, and organic solvent. Furthermore, proteins bound to nC-B resins can be efficiently eluted in neutral pH. Key performance parameters of nC-B resins and supporting results are summarized in Table 4.

**Table 4:** Key performance parameters of nC-B affinity chromatography resins

Attribute	Supporting results from test case with anti-SUMO nanoCLAMPs^a^
Efficient purification	Purification to > 90% in a single step
High dynamic binding capacity	240 nmol/ml (residence time of 1.2 min)
High thermal stability	T_m_ > 99 °C, resistant to boiling and autoclaving
High trypsin resistance	t_1/2_ >16 h in 0.1 mg/ml trypsin
High resistance to organic solvent	Full recovery of binding activity after 2 h in 100% DMF
High stability in NaOH	Full recovery of binding activity after > 20 cycles, each including 10 min incubation with 0.1 M NaOH
Activity-preserving options for elution buffers	100% yield after elution with imidazole pH 8 or citrate pH 2.5
Tolerance to oxidizing and reducing conditions	No cysteines or methionines in the framework sequence
Low-cost production	150–300 mg nanoCLAMP per liter of shaken *E. coli* culture

a Affinity resins based on Sulfolink crosslinked beaded agarose (Thermo).

This work provides a test case that supports the potential of nC-B resins to extend Protein A-like levels of performance to a broad range of proteins beyond antibodies. nC-B resins’ efficiency, ease of elution, low cost of manufacture, and reusability have the potential to reduce the total cost of manufacture for process-scale purifications. By way of comparison, other three loop single domain binders, such as nanobodies and single domain antibody VHH fragments, are routinely found that are comparable in affinity and specificity; however, to our knowledge, none have been described that withstand repeated NaOH treatment, organic solvent exposure, have melting temperatures above 80 °C, and are elutable at neutral pH. Traditional antibodies, with two sets of three complementarity determining regions (binding loops) per arm, while able to bind targets with unmatched affinity and specificity, are simply too large and fragile to withstand these harsh conditions.

The improved stability and performance of second-generation nanoCLAMPs also support their use in applications beyond affinity chromatography. Their general compatibility with high temperature, organic solvent, and pH extremes may enable industrial applications where extreme conditions are required. For example, nC-A class nanoCLAMPs have recently been utilized in electrochemical biosensors (23). The robustness of the improved nC-B class has the potential to enable the development of sensors and smart materials which encounter extreme conditions during manufacture or use.

The performance characteristics demonstrated for the exemplary nanoCLAMPs in this work support further exploration of nanoCLAMPs’ potential to serve as capture agents in a broad range of applications, especially those procedures requiring tight, selective, or reversible binding and exposure to extreme conditions.

## Experimental procedures

### Cloning of SMT3-A1 mutants

The plasmid pET(SMT3-A1) containing nanoCLAMP SMT3-A1 was mutated by inverse PCR (24) by amplifying the plasmid with forward and reverse primers containing the mutation(s) of interest with 15-bp overlapping 5′ ends, purifying the amplicon, In-Fusion cloning the ends back together (In Fusion HD Cloning Kit, Takara), and transforming chemically competent NEc1 *E. coli* (BL21(DE3) derivative with slyDΔ(His151-His196) from Nectagen, Inc). Plasmids were purified by Qiagen miniprep kit (Qiagen) and mutations were verified by sequencing the purified plasmids by Sanger sequencing (Genewiz). Glycerol stocks of the plasmids in NEc1 cells were prepared for seeding expression cultures. Constructs for conjugating to Sulfolink resin (Thermo) coded for the nanoCLAMP with an N-terminal 6-His tag and a 13-amino acid C-terminal GS-linker followed by a Cys. Constructs for expressing nanoCLAMPs for biophysical characterization lacked the GS-linker and the C-terminal Cys to avoid dimerization issues due to disulfides.

### Expression and purification of nanoCLAMPs under denaturing conditions for conjugation to affinity chromatography resin (1 L scale)

Glycerol stocks of NEc1 cells harboring nanoCLAMP expression vectors (described above) were used to inoculate 3 ml starter cultures of 2xYT/2% glucose, wt/vol (2% Glc)/100 μg/ml Carbenicillin (CB) and grown overnight at 37 °C, 250 rpm. The overnight cultures were diluted 1:100 into 300 ml of Novagen Overnight Express Instant TB Medium/1% glycerol/CB and incubated 24 h, 30 °C, 250 rpm. Cells were pelleted at 10,000*g*, 10 min, 4 °C, and lysed with 30 ml 100 mM NaH_2_PO_4_, 10 mM Tris, 6 M GuHCl (QAB) pH 8.5, plus 1 mM TCEP (QAB-TCEP, pH 8.5) using a Polytron to homogenize. The insoluble material was pelleted at 15,000*g*, 20 min, 15 °C, and the cleared supernatant applied to NiSeph6 FF (Cytiva) and incubated rotating for 1 h to overnight. The beads were transferred to a column and washed with 3 CV QAB-TCEP, pH 8.5, then 3 CV QAB, pH 8.5. The protein was eluted with QAB, pH 8.5 + 250 mM imidazole and quantified by A280. The purity of the eluted protein was measured by SDS-PAGE on 12% NuPAGE Bis-Tris gels and Coomassie staining with Gel-Code Blue (after removing the GuHCl by cold ethanol precipitation). Yields for nanoCLAMPs were typically 150 to 300 mg/L culture, and purity was typically greater than 90%.

The purified, denatured nanoCLAMPs in QAB, pH 8.5, were reduced with 2 mM TCEP if used after storage and conjugated to Sulfolink cross-linked, 6% beaded agarose (Thermo). Briefly, the resin was equilibrated with QAB, pH 8.5 + 5 mM EDTA and transferred to a column. The nanoCLAMP was adjusted to 8 mg/ml in volume 2× the volume of the Sulfolink resin and then incubated with the resin with rotation for 30 min at room temperature. The resin was allowed to settle for 15 min, and the column drained to the top of the resin bed. The column was washed with QAB, pH 8.5 and then incubated with 50 mM L-Cys in QAB, pH 8.5 to quench for 15 min with rotation. The column was allowed to settle, drained, and washed again with 6 M GuHCl, 20 mM Tris, (QCB) pH 8. Finally, the nanoCLAMP was refolded on the resin by rinsing with 6 CV of 20 mM MOPS, 150 mM NaCl (MBS), pH 6.5 + 1 mM CaCl_2_.

### Small-scale batch affinity chromatography and determination of the static binding capacity of nanoCLAMP resins

A spiked *E. coli* lysate was prepared by pelleting the equivalent of OD_600_ = 8 culture, discarding the supernatant, and lysing the cells with BPER at 4 ml per g pellet, 20 min at room temperature with rotation. The insoluble material was removed by centrifugation, and the cleared lysate adjusted to a total protein concentration of approximately 1.87 mg/ml, 20% BPER in PBS, pH 7.4. The target protein was spiked into the lysate to a final concentration of 0.025 to 0.2 mg/ml, depending on the application with a highly concentrated stock so that the total protein concentration remained unchanged. The spiked lysate was then incubated with 10 μl of the nanoCLAMP resin (packed volume) in a total volume of 1.4 ml, rotating at 4 °C for 1 h. The resin was precipitated by centrifugation and transferred to a small column. The resin was washed 4 times with 400 μl PBS, pH 7.4, and then eluted with 3 M imidazole, pH 8. The eluates were buffer exchanged twice with Zeba columns (7 kD MWCO, Thermo), quantified by A_280_ or fluorescence using an iD5 plate reader, and analyzed by SDS-PAGE as described above.

### Expression and purification of nanoCLAMPs for biophysical characterization

Glycerol stocks of NEc1 cells harboring nanoCLAMP expression vectors (described above) were used to inoculate 3 ml starter cultures of 2xYT/2% Glc/100 μg/ml CB and grown overnight at 37 °C, 250 rpm. The overnight cultures were diluted 1:100 into 35 ml of Novagen Overnight Express Instant TB Medium/1% glycerol/CB and incubated 24 h, 30 °C, 250 rpm. Cells were pelleted and lysed with QCB, pH 8, and insoluble material removed by centrifugation at 15,000*g*, 20 min, 15 °C. The cleared lysate was incubated with NiSeph6 FF (Cytiva) for > 1 h rotating, room temperature and then transferred to 2 ml columns. The columns were washed with 6 × 1 ml QCB, pH 8, then refolded with 11 ml of 20 mM MOPS, 150 mM NaCl (MBS), 1 mM CaCl_2_, pH 8. The nanoCLAMPs were eluted with MBS, 1 mM CaCl_2_, 250 mM imidazole, pH 8, buffer exchanged to remove the imidazole using Zeba seven MWCO desalting columns, and normalized to 1 mg/ml in MBS, 1 mM CaCl_2_, pH 6.5.

### Expression and purification of target proteins for panning, BLI, and affinity chromatography

To prepare a target protein for panning the library NL-26 and for biolayer interferometry experiments, we prepared a biotinylated yeast SUMO construct (B-SUMO, File S1) in a pET expression vector and transformed it into BL21(DE3) *E. coli* harboring a constitutively expressed biotin ligase, BirA. An overnight starter culture was diluted 1:100 into 500 ml Novagen Overnight Express Instant TB Medium/1% glycerol/CB/CAM including 5 mM biotin and incubated 24 h, 30 °C, 250 rpm. Following the induction, the cells were pelleted and the media discarded. The pellet was frozen at −80 °C, thawed on ice and resuspended at 5 ml/g pellet in MBS, pH 7.4 + Pierce Protease Inhibitor Tablet Mini and sonicated on ice for 10 min at 50% duty cycle. Biotin was added to 100 uM and the lysed cells incubated at 37 °C, 30 min, 250 rpm to drive biotinylation to completion. The lysate was cleared by centrifugation at 30,000*g*, 20 min, 4 °C and the supernatant transferred to a 2.25 ml SMT3-A1 resin (Nectagen, Inc) packed column at 1 ml/min. The resin was washed with 25 ml MBS, pH 7.4 and the protein eluted with polyol elution buffer (PEB): 10 mM Tris, 1 mM EDTA, 0.75 M ammonium sulfate, 40% propylene glycol, pH 7.9. The protein was desalted 2× into 50 mM Tris, pH eight, and stored as a 50% glycerol stock at −20 °C.

To facilitate the quantification of affinity chromatography target protein in eluates, we prepared a SUMO-GFP fusion (File S1) that we could track by fluorescence spectroscopy. Briefly, the pET expression construct was transformed into NEc1 *E. coli* (Nectagen, Inc) and an overnight culture diluted 1:100 into 1 L Novagen Overnight Express Instant TB Medium/1% glycerol/CB and grown 24 h at 30 °C, 250 rpm. The cells were pelleted, the media removed, and the cells lysed with BPER with Universal Nuclease (Thermo). The insoluble material was removed by centrifugation and the cleared supernatant was loaded onto a 5 ml NiSeph six FF column (Cytiva) at 1.5 ml/min, and the resin washed with 100 ml 50 mM NaH_2_PO_4_, 300 mM NaCl, 20 mM imidazole, pH 8, and then eluted with the same buffer with 250 mM imidazole. Protein purity of both B-SUMO and SUMO-GFP was assessed by SDS-PAGE using 12% NuPAGE, BisTris, MES running buffer under reducing conditions and stained with GelCode Blue (Thermo).

Target protein SUMO-NusA, which lacks a 6His tag, was expressed as described for SUMO-GFP but was purified using SMT3-A1(Resin) as described for B-SUMO, above.

The target proteins human CTLA-4, Fc tagged (Acro Biosystems Cat# CT4-H5255), and human TIGIT, Fc tagged (Acro Biosystems Cat#TIT-H5254), both expressed in HEK293 cells, were purchased from Acro Biosystems and spiked into indicated lysates at 0.1 mg/ml. The target protein SARS CoV 2 receptor binding domain (Wuhan strain) was purified from expi293 cells transiently transfected with expression vector NR52422 (BEI resources repository, https://www.niaid.nih.gov/research/bei-resources-repository) by affinity chromatography using nanoCLAMP P2710(Resin) (Nectagen, Inc) following a scaled-up version of the small scale batch affinity chromatography described above, substituting transfected expi293 supernatant for lysate, and spiked into non-transfected expi293 supernatant at 0.1 mg/ml.

### Analysis of monodispersity by size exclusion chromatography

Purified nanoCLAMPs were diluted to a final concentration of 0.18 mg/ml in MBS, 1 mM CaCl_2_, pH 6.5, centrifuged at 20,000*g*, 2 min 4 °C, and the supernatants transferred to a clean tube. The samples were loaded into a 125 μl sample loop and injected onto a Superdex 75 10/300 Gl column (GE Healthcare Life Sciences) equilibrated in MBS, 1 mM CaCl_2_, pH 6.5 at a flowrate of 0.65 ml/min. The column was calibrated with Bio-Rad Gel Filtration Standard per manufacturer’s instructions.

### Determination of melting temperature by differential scanning fluorimetry

The melting temperature of purified nanoCLAMPs was determined using GloMelt Thermal Shift Protein Stability Kit (Biotium) per the manufacturer’s instructions. Briefly, purified nanoCLAMPs were adjusted to 1 mg/ml in MBS, 1 mM CaCl_2_, pH 6.5 and diluted in half with 2× GloMelt (Biotium) and aliquoted to 386-well plate and sealed with optical film. The plate was then heated in a Quantstudio five qPCR machine using SYBR Green reporter with no passive reference. The heating profile was 25 °C for 2 min; ramp at 0.05 °C/sec to 99 °C; 99 °C for 2 min. T_m_ is defined as the inflection point in the unfolding curve.

### Determination of protease stability by digestion with trypsin and chymotrypsin

Digestions were performed by incubating the nanoCLAMPs at 0.25 mg/ml in a 20 μl reaction containing 0.1 mg/ml Trypsin (Roche Cat 11418475001) or Chymotrypsin (Roche Cat 11418467001) diluted in 1 mM HCl, such that the final HCl concentration in the reaction was 0.1 mM. CaCl_2_ was added to the reaction to 10 mM. The protein and remaining diluent buffer was MBS, pH 6.5. The reaction was incubated at 37 °C for the indicated times and stopped by adding 2 μl 10× Protease arrest (G-Biosciences), analyzed on SDS-PAGE (12% NuPAGE, Bis Tris, in MES buffer) in SDS sample buffer with reducing agent, and stained with GelCode Blue (Thermo). Densitometry was carried out using GelAnalyzer software to measure relative staining intensities of the full-length band.

### Phage display library NL-26 construction based on the nC-B class scaffold

The pCombX phagemid template p2799 (File S1) contained the N-terminal and C-terminal constant regions of the nC-B class separated by a stuffer region containing HindIII and SpeI cut sites. This template was digested with HindIII and SpeI, gel purified, and the plasmid region amplified with degenerate primers 1957T R and 1960T F, which added the N and C-terminal part of nC-B as well as the randomized loops V and Z, respectively. The primers listed with a T indicate they are degenerate primers constructed using phosphoramidite trimer mixes (Glen Research) of oligos (IDT) containing all amino acids except Cys, Met, Lys, and Arg. The short internal region of the P2788 was amplified using primers 1958T F and 1959 R, which added and randomized Loop W. PCR was carried out with ClonAmp HiFi PCR Mix, according to the manufacturer’s instructions (Takara Bio). The reaction cycle was 98 °C for 10 s, 65 °C for 10 s, and 72 °C for 30 s, repeated 30 times. These two amplicons, which contained overlapping ends, were gel purified and cloned together by Gibson Assembly (described below), creating the nC-B construct with three variable loops: Loop V (3 residues −817,819, 820), Loop W (7 residues, 838–844), and Loop Z (5 residues, 931–935), for a total of 15 variable residues in three loops.

To clone the library components, 10 μg of the large amplicon and 7.86 μg of the short amplicon were combined in a 2 ml reaction containing 1000 μl of Gibson Assembly Master Mix (2×) (NEB), and incubated at 50 °C for 30 min and then put on ice. The ligated DNA was then purified and concentrated in one Nucleospin Gel and PCR Cleanup Kit (Machery Nagel) and eluted in 45 μl EB. The DNA was then desalted on a VSWP 0.025 μm membrane (EMD Millipore) on ddH_2_O for 40 min with a water change at 20 min. The desalted DNA was then adjusted to 100 ng/μl with ddH_2_O and used to electroporate electrocompetent TG1 cells (Lucigen). Approximately 50 μl of DNA was added to 1.25 ml ice-cold TG1 cells and pipetted up and down 4 times to mix on ice, after which 25 μl aliquots were transferred to 50 electroporation cuvettes (with 1 mm gaps) on ice. The cells were electroporated, and immediately quenched with 975 μl recovery media (Lucigen), pooled, and incubated at 37 °C, 250 rpm for 1 h. To titer the library, 10 μl of recovered culture was serially diluted in 2xYT and 10 μl of each dilution spotted on 2xYT/2% Glc/CB and incubated at 30 °C overnight. The remaining library was expanded to 3 L 2xYT/2% Glc/CB and amplified overnight at 30 °C, 250 rpm. The next day, the library was pelleted at 10,000*g*, 10 min, 4 °C and the media discarded. The pellet was re-suspended to an OD_600_ of 75 in 2xYT/2% Glc/18% glycerol, aliquoted and stored at −80 °C.

### Panning of nanoCLAMP library NL-26 (nC-B library)

For the first round of panning, 2.7 L of 2xYT/2% Glc/CB was inoculated with 3.6 ml of the NL-26 library glycerol stock (OD_600_ = 75), to an OD_600_ of approximately 0.1 and grown at 37 °C, 250 rpm until the OD_600_ reached 0.52. The library was infected by adding helper phage VCSM13 (Stratagene, Cat#200251) to 750 ml of culture at an MOI of 20 phage/cell, and incubating at 37 °C, 100 rpm for 30 min, then 250 rpm for an additional 30 min. The cells were pelleted at 7500*g* for 10 min, and the media discarded. The cells were resuspended in 1.2 L 2xYT/CB, 70 μg/ml kanamycin (KAN), and incubated 15 h at 30 °C, 250 rpm. The cells were combined, and 100 ml was centrifuged at 10,000*g* for 10 min. The phage containing supernatant was transferred to clean tubes and precipitated by adding 37.5 ml of 5× PEG/NaCl (20% polyethylene glycol 6000/2.5 M NaCl), and incubated on ice for 25 min. The phage was pelleted at 13,000*g*, 25 min and the supernatant discarded. The phage was resuspended in 10 ml 20 mM NaH_2_PO_4_, 150 mM NaCl, pH 7.4 (PBS), then centrifuged at 15,000*g* for 15 min to remove insoluble material. The phage was precipitated a second time by adding ¼ volume 5× PEG/NaCl, incubated on ice for 5 min, and pelleted at 13,000*g*, 10 min at 4 °C. The phage pellet was resuspended in 3 ml PBS and quantified by absorbance at 268 nm (A_268_ = 1 for a solution of 5 × 10^12^ phage/ml).

Two sets of 100 μl of Dynabeads MyOne Streptavidin T1 (ThermoFisher Scientific) magnetic beads slurry were washed 2 × 1 ml with PBS-T (PBS with 0.05% Tween 20), applying magnet in between washes to remove the supernatant, and then blocked in 1 ml of 2% dry milk solution in PBS with 0.05% Tween 20 (2% M-PBS-T) for 1 h, rotating, at room temperature. To preclear the phage against beads alone, 1 ml of phage was prepared at a concentration of 2 × 10^13^ phage/ml in 2% M-PBS-T, the block removed from the first set of beads, and the phage added to the beads and incubated 1 h, rotating. The magnet was applied, and the precleared phage removed and transferred to a clean tube. The magnet was applied, and this step repeated two times to ensure no carryover of beads bound to phage to the next step. Biotinylated target (B-SUMO or other biotinylated target) was added to the precleared phage to 100 nM final concentration and incubated rotating 1 h. Block was removed from the second set of beads, and the phage/B-SUMO mix was added to the beads to precipitate the biotinylated target and bound phage. The beads were washed 8× with PBS-T, 1 ml each, vortexing between each step and applying the magnet. The washed beads were eluted with 800 μl 0.1 M glycine, pH 2.0, 10 min rotating, the magnet applied, and the eluate transferred to 72 μl 2 M Tris base to neutralize. The neutralized phage was then added to 9 ml XL1-blue *E. coli*, which had been grown to OD_600_ = 0.435 and placed on ice. The cells were infected at 37 °C, 45 min, 175 rpm, and then expanded to 100 ml 2xYT/2% Glc/CB and incubated overnight at 30 °C, 250 rpm.

The overnight cultures were harvested by measuring the OD_600_, centrifuging the cells at 10,000*g* for 10 min, and then resuspending the cells to an OD_600_ of 75 in 2xYT/18% glycerol. To prepare phage for the next round of panning, 5 ml of 2xYT/2% Glc/CB was inoculated with 5 μl of the 75 OD_600_ glycerol stock and incubated at 37 °C, 250 rpm until the OD_600_ reached 0.5. The cells were superinfected at 20:1 phage:cell, mixed well, and incubated at 37 °C, 30 min, 150 rpm and then 30 min at 250 rpm. The cells were pelleted at 5500*g*, 10 min, the glucose containing media discarded and the cells resuspended in 10 ml 2xYT/CB/KAN and incubated overnight at 30 °C, 250 rpm.

The overnight phage prep was processed as described above. The phage was then prepared at A_268_ = 0.8 in 2% M-PBS-T, and the panning and pre-clearing continued as described, except in the second and third rounds, the biotinylated target concentration was reduced 10× per round. Washes after phage capture were also increased in the third round, to 12 washes. In round 2, neutravidin-coated magnetic beads (Spherotech) were used in place of streptavidin beads to reduce enrichment for streptavidin binders.

### Qualitative semELISA of individual clones following panning

At the end of the last panning round, individual colonies were plated on 2xYT/2% Glc/CB agar plates following the 45 min 150 rpm recovery at 37 °C of the infected XL1-blue cells with the eluted phage. The next day, 95 colonies were inoculated into 400 μl 2xYT/2% Glc/CB in a 96-deep-well culture plate, and grown overnight at 37 °C, 300 rpm to generate a master plate, to which glycerol was added to 18% for storage at −80 °C. To prepare an induction plate for the ELISA, 5 μl of each master-plate culture was inoculated into 400 μl fresh 2xYT/0.1% Glc/CB medium and incubated for 2.75 h at 37 °C, 300 rpm. IPTG was then added to 0.5 mM and the plates incubated at 30 °C with 300 rpm shaking overnight. Because the phagemid contains an amber stop codon, some nanoCLAMP protein is produced without the pIII domain, although XL1-blue is a suppressor strain, resulting in the periplasmic localization of some nanoCLAMP, of which some percentage is ultimately secreted to the media. The media can then be used directly in an ELISA assay (soluble expression-based monoclonal enzyme-linked immunosorbent assay: semELISA). After the overnight induction, the plates were centrifuged at 1200*g* for 10 min to pellet the cells. Streptavidin coated microtiter plates (ThermoFisher) were rinsed 3 times with 200 μl PBS, and then coated with biotinylated target proteins at 2 μg/ml with 100 μl/well and incubated 1 h. For blank controls, a plate was incubated with 100 μl/well PBS. The coating solution was removed and the plates blocked with 2% M-PBS-T. The block was removed and 50 μl of 4% M-PBS-T added to each well. At this point 50 μl of each induction plate supernatant was transferred to the blank and protein-coated wells and pipetted 10 times to mix, and incubated 1 h. The plates were washed 4 times with 200 μl PBS-T and the plates dumped and slapped on paper towels in between washes. After the washes, 75 μl of 1:2000 dilution anti-FLAG-HRP (Sigma A8592) in 4% M-PBS-T was added to each well and incubated 1 h. The anti-FLAG-HRP was discarded and the plates washed as before. The plates were developed by adding 75 μl TMB Ultra substrate (ThermoFisher), and analyzed for positive signals compared to controls. Positive clones were then grown up from the master plate by inoculating 1 ml 2×YT/2% Glc/100 μg/ml CB with 3 μl glycerol stock and incubated for at least 6 h at 37 °C, 250 rpm. The cells were then pelleted and the media discarded. Plasmid DNA was prepared from the pellets using the Qiaprep Spin Miniprep Kit, and the sequences determined by Sanger sequencing at Genewiz. The nanoCLAMP inserts from unique positive clones were amplified and cloned into the pET expression vector, described above.

### Biolayer interferometry of nanoCLAMPs

Kinetic analysis of interactions between nanoCLAMPs and biotinylated SUMO was carried out on an OctetRed96 using SAX streptavidin-coated sensor tips. In one experiment, biotinylated SMT3-A1 was immobilized and non-biotinylated SUMO-NusA was used as an analyte. The tips were transferred first to buffer (MBS, 1 mM CaCl_2_, pH 6.5 + 1% BSA) for 300 s, then to B-SUMO at 2 μg/ml in the buffer for 180 s, then to buffer for 300 s, then to at least four dilutions of nanoCLAMPs in buffer (association) for 200 or 600 s, then to buffer (dissociation) for 400 to 500 s. The cells were constantly vortexing at 1000 rpm at rm temp. The sensorgrams were fit to a 1:1 model and K_d_ calculated using global fit analysis.

### Dynamic binding capacity of P2808 resin and SMT3-A1 resin

A packed volume of 0.6 ml of P2808 resin or SMT3-A1 resin was packed into a Tricorn 5/50 column (5 mm ID × 3.06 cm height) and equilibrated in 20 mM NaH_2_PO_4_, 150 mM NaCl, pH 7.4 (PBS) at 0.5 ml/min for 5 CV. The load, a Sumo-GFP fusion protein (MW = 41,559 g/mol) diluted to a concentration, c, of 0.2 mg/ml in PBS, was pumped through the system with the column on bypass and the eluate fluorescence measured to determine the total load fluorescence at Ex/Em 485/535 nm. The delay volume, V_delay_, was measured for the configuration at 0.5 ml. The load was then directed to the column and the volume V_x_ measured, where V_x_ is the volume where the fluorescence of the eluate = 10% of that of the total load. The dynamic binding capacity, in units of mg/(mL resin), was then calculated as follows: DBC = (V_x_ – V_delay_)∗c/(Vol Resin).

### Purification of Sumo-GFP from a spiked *E. coli* lysate by affinity chromatography with P2808 resin

A cleared *E. coli* lysate was prepared by lysing a pellet of NEc1 *E. coli* with BPER (Thermo) and removing insoluble material by centrifugation at 15,000*g*, 20 min, 4 °C. The cleared supernatant was diluted to a total protein concentration of roughly 3.3 mg/ml with PBS, pH 7.4 such that the BPER reagent was present at 20% vol/vol. The target protein SUMO-GFP (MW = 41,559 g/mol) was spiked-in to a final concentration of either 0.2 mg/ml or 0.025 mg/ml. The spiked lysate was loaded onto the column at 0.5 ml/min for indicated times, washed with 20 CV PBS, pH 7.4, then eluted with 3 M Imidazole, pH 8. Fractions containing eluted target were pooled and desalted 2× on Zeba seven MWCO columns and the protein quantified by A_280_. Imidazole removal was verified by testing the A280 of elution buffer alone following 2× desalting. Spiked lysate, early wash fractions, and pooled elutions (post buffer exchange) were analyzed by NuPAGE SDS PAGE under reducing conditions, 12%, Bis-Tris in MES running buffer and stained with Gel Code Blue (Thermo).

### Repeated AC purification cycles including cleaning in place of resins using 0.1 M NaOH

Repeated affinity chromatography purifications were carried out on an FPLC with a small 50 μl (packed) column using running buffer 20 mM MOPS, 150 mM NaCl, 1 mM CaCl_2_, pH 7.2. The Load consisted of Sumo-GFP spiked into a cleared *E. coli* lysate (described above in Purification of Sumo-GFP from a spiked *E. coli* lysate) at 0.1 mg/ml. The cycle consisted of a 2 ml equilibration in running buffer at 1 ml/min, 0.5 ml load of spiked lysate at 0.5 ml/min, 3 ml wash with running buffer at 0.5 ml/min, 2 ml elution with 3 M imidazole, pH 8 (collected) at 0.5 ml/min, a 0.5 ml wash with running buffer at 0.5 ml/min, a cleaning in place cycle of 1.5 ml NaOH at 1 ml/min and then 2 ml at 0.2 ml/min (total contact time 10 min), and finally a refolding step with 5 ml running buffer at 1 ml/min. The target concentration in the eluates was measured by fluorescence spectroscopy in duplicate on an iD5 plate reader (Molecular Dynamics) Ex/Em 485/535 nm. The eluates were analyzed by SDS-PAGE using NuPAGE gels as described above.

### Repeated AC purification cycles with low pH elution and short cleaning in place with NaOH

Repeated affinity chromatography purifications were carried out as described above, except the column was eluted with 0.1 M citrate, pH 2.5 instead of 3 M imidazole, pH 8. Also, the cleaning in place step with 0.1 M NaOH was shortened to 1 ml, at 1 ml/min (1 min contact time per cycle). Since the eluted SUMO-GFP was denatured by the low pH elution, the relative elution concentrations were compared using densitometry of the target bands on SDS PAGE.

### Determination of effect of autoclaving or DMF incubation on SUMO binding resin binding capacity and specificity

For each resin tested, three 10 μl aliquots (packed vol) of resin were loaded into 1.5 ml screw-cap tubes. To one, 1 ml DMF was added, and the tube incubated at rm temp for 2 h. To the other two, 100 μl MBS, 1 mM CaCl_2_, pH 7.2 was added. One of these was autoclaved with its cap left slightly loose, on a 30 min liquid cycle, which sterilizes at around 120 °C and 20 psi for 30 min, and then slowly drops the pressure and the temperature over the next 90 min. The other set of resin was left on ice as a control. After 2 h all of the resins were cooled to room temperature and centrifuged at 1000*g*, 1 min. The control and autoclaved resin were stored overnight at 4 °C. The DMF treated resin was rinsed 3× with fresh MBS, 1 mM CaCl_2_, pH 7.2 and then stored overnight at 4 °C. The next day all three of the sets of beads were rinsed with fresh buffer and then incubated with 1.3 ml of *E. coli* lysate (prepared as described above) spiked with SUMO-GFP at 0.2 mg/ml, for 1 h, 4 °C, rotating. The resin was loaded into a small, tared column, rinsed 4 × 400 μl PBS, pH 7.4, then eluted 3 × 25 μl 3 M imidazole, pH 8. The fluorescence of the eluates was read on an iD5 plate reader in duplicate as described and the concentration determined by comparison with a standard curve of the target and compared to controls. The concentrations were normalized and analyzed by SDS PAGE as described earlier to assess purity.

## Data availability

All data shown is available in this paper.

## Supporting information

This article contains supporting information.

## Conflict of interest

The authors declare the following financial interests/personal relationships which may be considered as potential competing interests: RJS and DMC are significant shareholders of Nectagen, Inc.

## Figures and Tables

**Figure:**
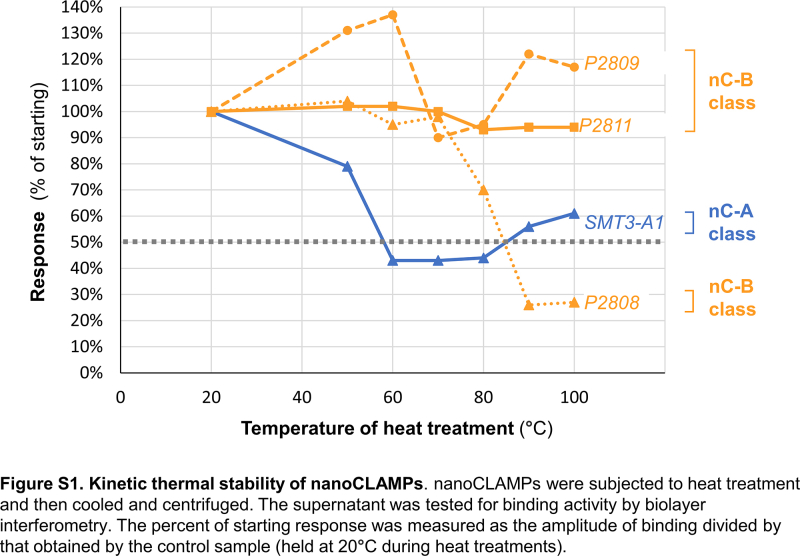
S1

**Fig:**
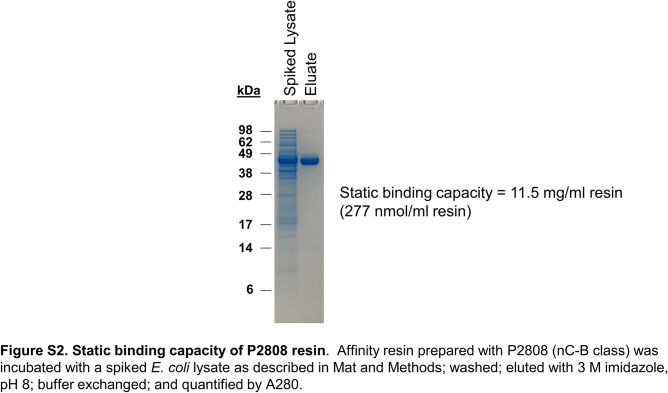
S3

**Fig:**
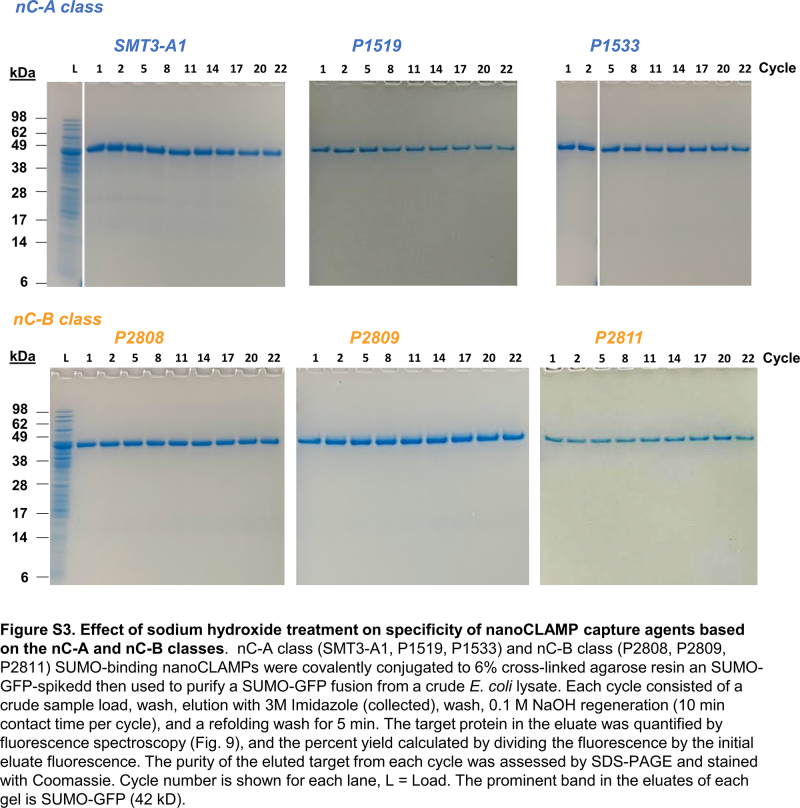
S2

**Fig:**
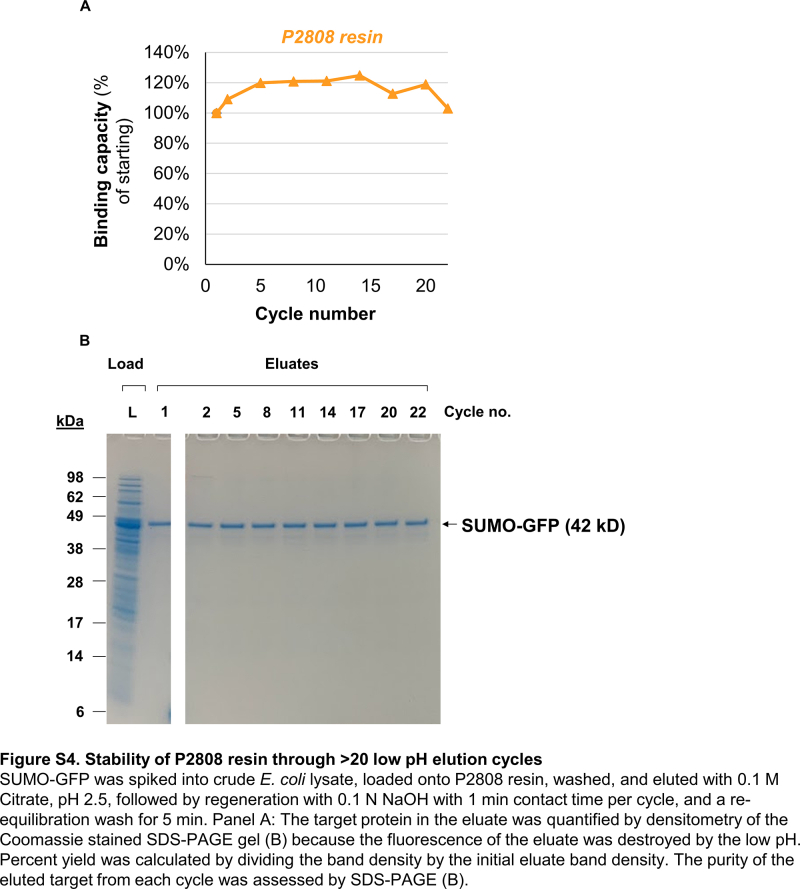
S4

**Fig:**
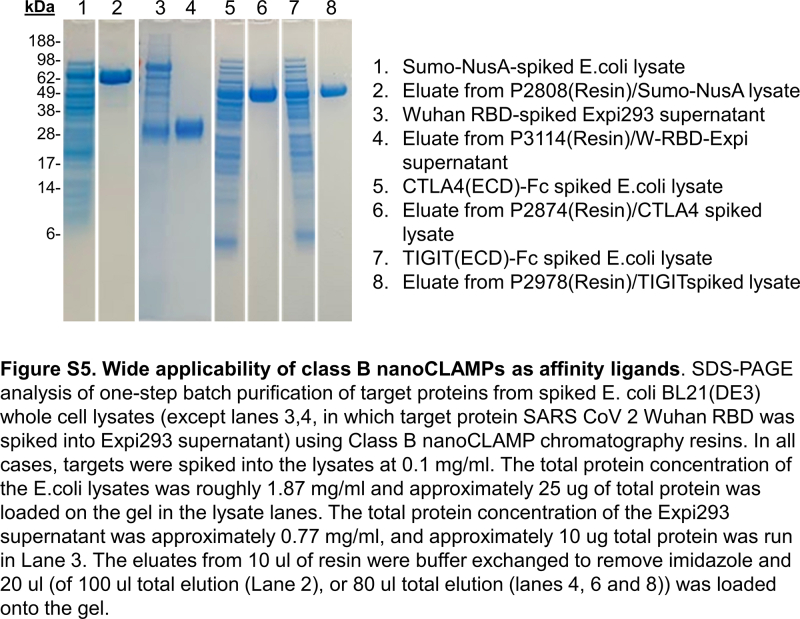
S5

**Fig:**
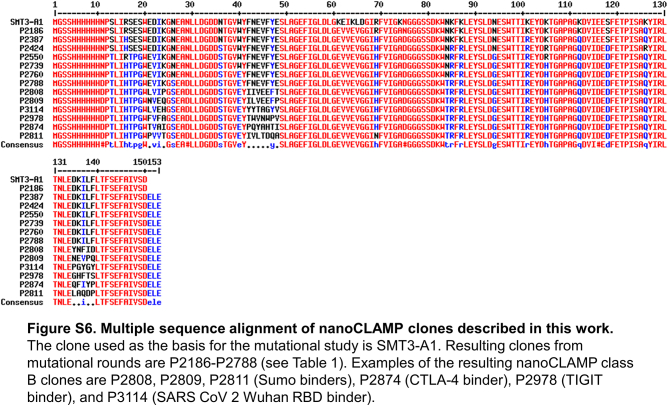
S6

**Fig:**
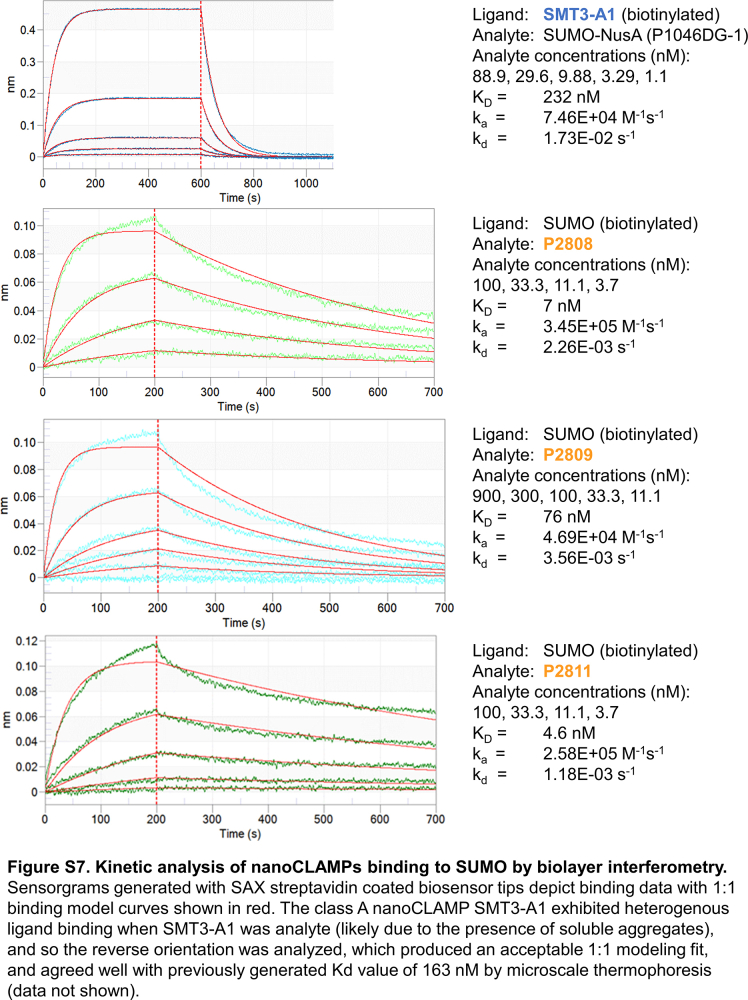
S7
