# Supplementary material for: Protein engineering of a nanoCLAMP antibody mimetic scaffold as a platform for producing bioprocess-compatible affinity capture ligands
Source: J Biol Chem. 2023 Jun 12;299(7):104910. doi: 10.1016/j.jbc.2023.104910 (PMC10404686; doi:10.1016/j.jbc.2023.104910)
Supplement: Table S1 [file mmc3.docx]

**Supporting Information Table S1. Orthologous sequences used for consensus-based design**

| **Species of origin** | **Homology to *C. perfringens* CBM32-2 residues 808-943** (percent identity) |
| --- | --- |
| *Clostridium nigeriense* | 58% |
| *Clostridium tertium* | 52% |
| *Clostridium* sp. C8 | 50% |
| *Clostridium* sp. 7_2_43FAA | 53% |
| *Clostridium chauvoei* | 50% |
| *Clostridium paraputrificum* | 52% |
| *Clostridium celatum* | 51% |
| *Clostridium multispecies* | 51% |
| *Clostridium septicum* | 47% |
| *Clostridium spiroforme* | 45% |
| *Erysipelatoclostridium ramosum* | 46% |
| *Bacillus mesonae* | 44% |
| *Tyzzerella* sp. An114 | 44% |
| *Bacillus* sp. B-jedd | 42% |
| *Bacillus niacini* | 41% |
| *Bacillus mesonae* | 40% |
| *Sporosarcina* sp. ZBG7A | 39% |
| *Sporosarcina* sp. D27 | 40% |
| *Lysinibacillus sphaericus* | 40% |
| *Coprobacillus* sp. AF21-8LB | 43% |

**Supporting Information Table S3. Summary of T_m_ and monodispersity data for sets of nanoCLAMPs to three different target proteins**

| **nC group from Table S2** | **# binders** | **T_m_ median (°C) by DSF analysis** | **% > 75% monomer (by SEC analysis)** |
| --- | --- | --- | --- |
| SUMO binders | 18 | 79 | 67% |
| Target 1 binders | 22 | 78 | 45% |
| Target 2 binders | 39 | 71 | 62% |
